# Potent, selective, and subunit‐dependent activation of TRPC5 channels by a xanthine derivative

**DOI:** 10.1111/bph.14791

**Published:** 2019-09-06

**Authors:** Aisling Minard, Claudia C. Bauer, Eulashini Chuntharpursat‐Bon, Isabelle B. Pickles, David J. Wright, Melanie J. Ludlow, Matthew P. Burnham, Stuart L. Warriner, David J. Beech, Katsuhiko Muraki, Robin S. Bon

**Affiliations:** ^1^ School of Chemistry University of Leeds Leeds UK; ^2^ Department of Discovery and Translational Science, Leeds Institute of Cardiovascular and Metabolic Medicine University of Leeds Leeds UK; ^3^ Discovery Sciences, R&B Biopharmaceuticals AstraZeneca Alderley Park UK; ^4^ Laboratory of Cellular Pharmacology, School of Pharmacy Aichi‐Gakuin University Nagoya Japan

## Abstract

**Background and Purpose:**

The TRPC1, TRPC4, and TRPC5 proteins form homotetrameric or heterotetrameric, calcium‐permeable cation channels that are involved in various disease states. Recent research has yielded specific and potent xanthine‐based TRPC1/4/5 inhibitors. Here, we investigated the possibility of xanthine‐based activators of these channels.

**Experimental Approach:**

An analogue of the TRPC1/4/5 inhibitor Pico145, AM237, was synthesized and its activity was investigated using HEK cells overexpressing TRPC4, TRPC5, TRPC4–C1, TRPC5–C1, TRPC1:C4 or TRPC1:C5 channels, and in A498 cells expressing native TRPC1:C4 channels. TRPC1/4/5 channel activities were assayed by measuring intracellular concentration of Ca^2+^ ([Ca^2+^]_i_) and by patch‐clamp electrophysiology. Selectivity of AM237 was tested against TRPC3, TRPC6, TRPV4, or TRPM2 channels.

**Key Results:**

AM237 potently activated TRPC5:C5 channels (EC_50_ 15–20 nM in [Ca^2+^]_i_ assay) and potentiated their activation by sphingosine‐1‐phosphate but suppressed activation evoked by (−)‐englerin A (EA). In patch‐clamp studies, AM237 activated TRPC5:C5 channels, with greater effect at positive voltages, but with lower efficacy than EA. Pico145 competitively inhibited AM237‐induced TRPC5:C5 activation. AM237 did not activate TRPC4:C4, TRPC4–C1, TRPC5–C1, TRPC1:C5, and TRPC1:C4 channels, or native TRPC1:C4 channels in A498 cells, but potently inhibited EA‐dependent activation of these channels with IC_50_ values ranging from 0.9 to 7 nM. AM237 (300 nM) did not activate or inhibit TRPC3, TRPC6, TRPV4, or TRPM2 channels.

**Conclusions and Implications:**

This study suggests the possibility for selective activation of TRPC5 channels by xanthine derivatives and supports the general principle that xanthine‐based compounds can activate, potentiate, or inhibit these channels depending on subunit composition.

**Abbreviations**
[Ca^2+^]_i_: intracellular concentration of Ca^2+^
EA: (−)‐englerin A
HEK T‐REx cells: HEK 293 cells overexpressing tetracycline‐inducible recombinant proteins

**What is already known**

- Xanthines such as Pico145 and HC‐070 are (sub)nanomolar, highly selective inhibitors of TRPC1/4/5 channels.
- Xanthine‐based inhibitors of TRPC1/4/5 channels are orally bioavailable and suitable for in vivo studies.

**What this study adds**

- The xanthine AM237 potently and selectively activates homomeric TRPC5 channels but inhibits other TRPC1/4/5 channels.
- Pico145 is a competitive inhibitor of AM237 in the context of homomeric TRPC5 channels.

**What is the clinical significance**

- AM237 may allow the identification of TRPC1/4/5 subunit composition in clinical samples.
- This study contributes to the understanding of the mode‐of‐action of xanthine‐based TRPC1/4/5 channel modulators.

## 1 INTRODUCTION

The transient receptor potential canonical (TRPC) proteins are members of the TRP superfamily, which also includes TRPV, TRPM, and TRPA (Abramowitz & Birnbaumer, 2009; Bon & Beech, 2013; Montell, 2005; Montell et al., 2002; Venkatachalam & Montell, 2007; Voets, Talavera, Owsianik, & Nilius, 2005). There are seven TRPC subtypes (Montell, 2005), of which all but http://www.guidetopharmacology.org/GRAC/ObjectDisplayForward?objectId=487 are expressed in humans (Vannier et al., 1999; Wes et al., 1995; Zhu et al., 1996). The human TRPCs can be further divided into two clusters—http://www.guidetopharmacology.org/GRAC/ObjectDisplayForward?objectId=486/http://www.guidetopharmacology.org/GRAC/ObjectDisplayForward?objectId=489/http://www.guidetopharmacology.org/GRAC/ObjectDisplayForward?objectId=490 and http://www.guidetopharmacology.org/GRAC/ObjectDisplayForward?objectId=488/http://www.guidetopharmacology.org/GRAC/ObjectDisplayForward?objectId=491/http://www.guidetopharmacology.org/GRAC/ObjectDisplayForward?objectId=492—based on sequence homology (Clapham, Runnels, & Strübing, 2001). TRPC proteins assemble as tetramers to form Ca^2+^‐permeable, non‐selective cation channels, which may consist of homomers or heteromers of subunits, each with their own characteristics and functions. The TRPC proteins are widely expressed in excitable and non‐excitable cells, and TRPC channels are modulated by many physiological mechanisms and chemical and physical factors, including G‐protein signalling, receptor tyrosine kinase signalling, temperature, heavy metal ions, lipids, and dietary compounds. This has led to the idea that the channels are complex integrators of multiple physiological and environmental stimuli (Bon & Beech, 2013; Clapham, 2003; Mederos y Schnitzler, Gudermann, & Storch, 2018; Naylor et al., 2016; Storch et al., 2017).

TRPC4 and TRPC5 are the most closely related TRPC proteins (Plant & Schaefer, 2003; 70% sequence identity; BLAST search; Altschul, Gish, Miller, Myers, & Lipman, 1990). In this paper, we refer to homomeric channels as TRPC4:C4 or TRPC5:C5 and to heteromeric channels as TRPC1:C5 or TRPC1:C4. The term TRPC1/4/5 is more general, denoting channels composed of TRPC1, TRPC4 and/or TRPC5, as homomers or heteromers in any ratio. A final notation used here is TRPC4–C1 and TRPC5–C1 which denotes channels composed of recombinant, concatemeric proteins (fusions of TRPC1 at the C‐terminus of either TRPC4 or TRPC5 through a short linker; Ludlow et al., 2017; Rubaiy, Ludlow, Henrot, et al., 2017; Rubaiy, Ludlow, Bon, & Beech, 2017). We use similar notations for TRPC3/6/7 channels.

Cryo‐EM structures of mouse TRPC4:C4 (Duan et al., 2018), zebrafish TRPC4:C4 (Vinayagam et al., 2018), and mouse TRPC5:C5 (Duan et al., 2019) have recently been reported. However, the precise native stoichiometries of TRPC1/4/5 channels are largely unknown (Minard et al., 2018). Overexpression of TRPC4 or TRPC5 protein leads to functional homotetramers, whereas the TRPC1 protein may not form functional homomeric channels but is widely expressed and an important contributor to heteromeric channels, for example, with TRPC4 and/or TRPC5 (Akbulut et al., 2015; Bon & Beech, 2013; Bröker‐Lai et al., 2017; Dietrich, Fahlbusch, & Gudermann, 2014; Ludlow et al., 2017; Minard et al., 2018; Sukumar et al., 2012).

Although observational clinical studies and changes detected in genetically or pharmacologically modified rodents and/or human tissue suggest multiple physiological roles of TRPC1/4/5 channels (Bon & Beech, 2013; Bröker‐Lai et al., 2017; Lau et al., 2016; Lepannetier et al., 2018), disruption of the *Trpc4/5* genes (Suresh Babu, Wojtowicz, Birnbaumer, Hecker, & Cattaruzza, 2012) and global expression of a dominant‐negative mutant TRPC5 (Sukumar et al., 2012) do not cause catastrophic phenotypes. However, the involvement of TRPC1/4/5 channels in various human diseases—including CNS disorders, kidney disease, cancer, cardiovascular disease, and complications of diabetes—has led these channels to emerge as potential therapeutic targets (Bon & Beech, 2013; Gaunt, Vasudev, & Beech, 2016; Just et al., 2018; Minard et al., 2018; Zhou et al., 2017).

**Figure 1:**
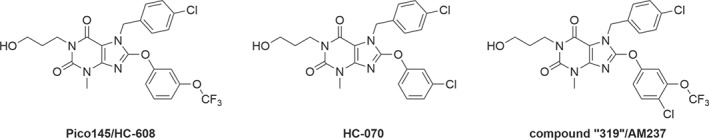
Structures of Pico145, HC‐070, and AM237

Several highly potent and selective TRPC1/4/5 modulators have been described recently (Minard et al., 2018). Currently, the most potent TRPC1/4/5 activator is the natural product http://www.guidetopharmacology.org/GRAC/LigandDisplayForward?ligandId=8372 (EA; Akbulut et al., 2015; Carson et al., 2015; Ludlow et al., 2017; Muraki et al., 2017), which induces strong TRPC1/4/5 currents at nanomolar concentrations. However, its toxicity and instability in rodent serum and in the gastrointestinal tract limit its use for in vivo studies (Carson et al., 2015; Cheung et al., 2018). Other promising TRPC1/4/5 activators include the natural product http://www.guidetopharmacology.org/GRAC/LigandDisplayForward?ligandId=10287 (Rubaiy et al., 2018), the marketed drug http://www.guidetopharmacology.org/GRAC/LigandDisplayForward?ligandId=2326 (which has limited potency and selectivity but has been used for in vivo studies of TRPC5 channels; Richter, Schaefer, & Hill, 2014; Zhou et al., 2017; Zhu et al., 2018), and the synthetic benzothiadiazine derivative http://www.guidetopharmacology.org/GRAC/LigandDisplayForward?ligandId=10289, which activates TRPC5:C5 (EC_50_ ~1 μM), TRPC1:C5, and TRPC4:C5 channels, but not TRPC4:C4 or TRPC1:C4 channels (Beckmann et al., 2017). The most promising TRPC1/4/5 inhibitors are two closely related xanthines, http://www.guidetopharmacology.org/GRAC/LigandDisplayForward?ligandId=10291 (also called HC‐608) and http://www.guidetopharmacology.org/GRAC/LigandDisplayForward?ligandId=10286 (Figure 1; Just et al., 2018; Rubaiy, Ludlow, Bon, & Beech, 2017; Rubaiy, Ludlow, Henrot, et al., 2017), which were originally described in a patent (Chenard & Gallaschun, 2014). These compounds exhibit (sub)nanomolar potency against TRPC1/4/5 channels, while at 1–2 μM, no significant effects on hundreds of other targets were found (Just et al., 2018). The compounds are orally bioavailable and suitable for in vivo studies (Cheung et al., 2018; Just et al., 2018). Pico145 also distinguishes between different TRPC1/4/5 tetramers, with the highest potency against heteromeric channels (Rubaiy, Ludlow, Henrot, et al., 2017).

The mechanism by which Pico145 and HC‐070 inhibit TRPC1/4/5 channel function is not known, but based on outside‐out patch‐clamp recordings, Pico145 is thought to act via a distinct binding site accessible from the extracellular side of the plasma membrane (Rubaiy, Ludlow, Henrot, et al., 2017). Although quantitative data were not provided, the patent describing Pico145 and HC‐070 suggested that a small number of xanthine derivatives may act as TRPC5 activators instead of inhibitors (Chenard & Gallaschun, 2014). One of these, compound “319,” differs from Pico145 by only a single chloride substituent (Figure 1). Because of the paucity of potent TRPC1/4/5 activators suitable for in vivo studies, and in order to learn more about the remarkable properties of xanthine‐based TRPC1/4/5 modulators, we decided to synthesize compound “319” (which we named http://www.guidetopharmacology.org/GRAC/LigandDisplayForward?ligandId=10421) and conduct studies of its effects on homomeric and heteromeric TRPC1/4/5 channels.

## 2 METHODS

### 2.1 Plasmids

To facilitate cloning of TRPC5‐SYFP2 and TRPC4‐SYFP2, a pcDNA™4/TO expression vector (ThermoFisher Scientific, Waltham, MA, USA), into which a four amino acid linker (ASAS) flanked by AgeI and SacII restriction sites had been introduced between EcoRI and XhoI restriction sites, was used (Naylor et al., 2016). Human TRPC4β was inserted between BamHI and AgeI restriction sites as described previously (Ludlow et al., 2017). Human TRPC5 (forward primer: 5′ GCTTGGTACCGCCACCATG 3′ and reverse primer: 5′ TGACACCGGTGAGGCGAGTTGTAACTTGTTCTTC 3′) was inserted upstream of the linker between KpnI and AgeI restriction sites using hTRPC5/pcDNA™4/TO (Zeng et al., 2004) as a PCR template. Both constructs contained an N‐terminal Kozak sequence. SYFP2 was inserted downstream of the linker between SacII and XbaI restriction sites using pSYFP2‐C1 as a PCR template (forward primer, 5′ ATAACCGCGGAATGGTGAGCAAGGGCGAG 3′; reverse primer, 5′ ATGTTCTAGATTACTTGTACAGCTCGTCCATGC 3′). pSYFP2‐C1 was a gift from Dorus Gadella (Addgene plasmid #22878; http://n2t.net/addgene:22878; RRID:Addgene_22878; Kremers, Goedhart, van Munster, & Gadella, 2006). Plasmids were sequenced to verify identity.

### 2.2 Cell culture and expression systems

HEK T‐REx™ cell line was transfected with TRPC5‐SYFP2 or TRPC4‐SYFP2 using FuGENE® HD transfection reagent. After 48 hr, cells were put under antibiotic selection using 400 μg·ml^−1^ zeocin and 10 μg·ml^−1^ blasticidin S (Invivogen, San Diego, California, USA). Medium changes were carried out regularly to remove dead cells. HEK T‐REx cells expressing tetracycline‐inducible human TRPC5, TRPC4–C1, and TRPC5–C1 were created as described previously (Akbulut et al., 2015; Ludlow et al., 2017; Rubaiy, Ludlow, Henrot, et al., 2017). TRPC5–C1 and TRPC4–C1 concatemers allow expression of functional channels with defined 2:2 stoichiometry that display ion permeability and current–voltage relationships distinct from those of homomeric TRPC4:C4 or TRPC5:C5 channels and consistent with those obtained upon co‐expression of TRPC1 with TRPC4 or TRPC5 (Ludlow et al., 2017; Rubaiy, Ludlow, Bon, & Beech, 2017; Rubaiy, Ludlow, Henrot, et al., 2017). Cells were cultured in DMEM‐F12 GlutaMAX (Invitrogen, Paisley, UK) containing 10% FBS (Sigma‐Aldrich, Gillingham, UK), with the addition of blasticidin (10 μg·ml^−1^) and zeocin (400 μg·ml^−1^) to maintain the stable incorporation of the tetracycline repressor and the channel of interest, respectively. Expression of proteins was induced by the addition of tetracycline (1 μg·ml^−1^, 24 hr). Cells were maintained in a humidified incubator with 5% CO_2_ at 37°C. HEK 293 cells stably expressing tetracycline‐regulated human http://www.guidetopharmacology.org/GRAC/ObjectDisplayForward?objectId=494 were prepared similarly and have also been described previously (McHugh, Flemming, Xu, Perraud, & Beech, 2003). For experiments using transient co‐expression of TRPC1 and TRPC5, HEK cells at 40–60% confluency were transfected with pIRES2‐AcGFP1 and pIRES2‐DsRed‐Express2 plasmids containing human TRPC1 and TRPC5, respectively, using Lipofectamine 3000 (ThermoFisher Scientific, Yokohama, Japan). All experiments using transient expression were performed within 48 hr after transfection. A498 cells were cultured in Minimum Essential Medium containing Earle's salts (ThermoFisher Scientific, Loughborough, UK), supplemented with 10% FBS and penicillin–streptomycin (100 units per ml^−1^, 100 μg·ml^−1^; ThermoFisher Scientific, Loughborough, UK). CHO cells stably expressing http://www.guidetopharmacology.org/GRAC/ObjectDisplayForward?objectId=510 were maintained in Ham's Nutrient Mixture F12 (ThermoFisher Scientific, Loughborough, UK) supplemented with 10% FBS, penicillin–streptomycin, and 1 mg·ml^−1^ G418 (InvivoGen, San Diego, California, USA). For experiments using hTRPC3 and hTRPC6, WT HEK 293 cells were transfected with hTRPC3 (Naylor et al., 2016) or hTRPC6 (cloned into pcDNA3) using jetPRIME® transfection reagent (VWR, Lutterworth, UK). Cells were assayed 48 hr after transfection.

### 2.3 Intracellular Ca^2+^ measurements

Intracellular concentration of Ca^2+^ ([Ca^2+^]_i_) recordings were carried out using the ratiometric Ca^2+^ dye Fura‐2; 24 hr before experiments, cells were plated onto black, clear bottom poly‐d‐lysine coated 96‐well plates, and for T‐REx cells, TRPM2 or TRPC1/4/5 protein expression was induced with 1 μg·ml^−1^ tetracycline at this point. All cells were used at 90% confluence. To load cells with the Fura‐2 dye, media was removed and cells were incubated with standard bath solution (SBS; composition, in mM, NaCl 130, KCl 5, glucose 8, HEPES 10, MgCl_2_ 1.2, and CaCl_2_ 1.5) containing 2‐μM Fura‐2 acetoxymethyl ester (Fura‐2 AM) and 0.01% pluronic acid for 1 hr at 37°C. For CHO cells stably expressing TRPV4 protein, probenecid (final concentration 2.5 mM) was present in all buffers used from the point of Fura‐2 AM application. After this incubation, cells were washed with fresh SBS and incubated at room temperature for a further 30 min. SBS was then changed to recording buffer (SBS with 0.01% pluronic acid and 0.1% DMSO to match compound buffer) immediately prior to experimentation. In the case of inhibitor studies, cells were washed twice with fresh SBS after incubation with Fura‐2 AM, followed by the addition of SBS with 0.01% pluronic acid and the relevant inhibitor or vehicle. Cells were then incubated for 30 min prior to the experiment. In the case of Ca^2+^‐free recordings, both recording buffer and compounds were made up in Ca^2+^‐free SBS, where CaCl_2_ was replaced by 0.4‐mM EGTA. Measurements were carried out using a FlexStation (Molecular Devices, San Jose, CA), using excitation wavelengths of 340 and 380 nm, at an emission wavelength of 510 nm. [Ca^2+^]_i_ recordings were performed at room temperature at 5 s intervals for 300 s (unless stated otherwise). Compounds were added from a compound plate after recording for 60 s.

### 2.4 Cell viability assay

Cell viability was assayed using the LIVE/DEAD® cell viability kit (Molecular Probes™, Eugene, Oregon, USA). HEK T‐REx expressing tetracycline‐inducible hTRPC5 were plated onto black, clear bottom poly‐d‐lysine‐coated 96‐well plates, at 25,000 cells per well, and TRPC5 protein expression was induced with 1 μg·ml^−1^ tetracycline. Cells were cultured for 24 hr in DMEM‐F12 GlutaMAX (Invitrogen, Paisley, UK) containing 10% FBS, with the addition of blasticidin (10 μg·ml^−1^) and zeocin (400 μg·ml^−1^). For wells containing methanol‐treated positive control cells, medium was removed and 70% methanol/distilled water (100 μl) was added and cells returned to the incubator for 30 min. After incubation, the solution in each well was replaced with recording buffer (SBS with 0.01% pluronic acid and 0.05% DMSO; 100 μl). The LIVE/DEAD® (Molecular Probes™, Eugene, Oregon, US) cell viability kit was used to stain the cells. Calcein AM (5 μl) and ethidium homodimer‐1 (20 μl) were added to SBS with 0.01% SBS (10 ml). Compound and vehicle dilutions were carried out directly in LIVE/DEAD® solution and 100 μl of each was added to the recording buffer in each corresponding well. Cells were incubated for 30 min and immediately imaged. Imaging and analysis was carried out on the IncuCyte® ZOOM (Essen Bioscience, Welwyn Garden City, UK).

### 2.5 Patch‐clamp recording

For electrophysiology experiments, cells were plated at a low density of 20–30% onto round or square glass coverslips (13 mm diameter or 5 × 5 mm), and TRPC expression in HEK T‐REx was induced with 1 μg·ml^−1^ tetracycline 24 hr before experimentation. Experiments were carried out at room temperature. Extracellular solution consisted of SBS. Patch‐clamp recordings were performed in whole‐cell mode (Figure S7) and in excised outside‐out patch mode under voltage clamp at room temperature using 2–4 MΩ patch pipettes fabricated from borosilicate glass capillaries with an outside diameter of 1 mm and an inside diameter of 0.58 mm (Harvard Apparatus). The voltage protocol comprised voltage ramps applied from −100 to +100 mV every 10 s from a holding potential of 0 mV. The patch‐clamp currents were recorded using Axopatch 200B or HEKA EPC‐800 amplifiers, digitized by a Digidata 1440 or PCI6229 (National Instruments Japan, Tokyo, Japan) and recorded to a computer using pCLAMP10 (Molecular Devices) or WinWCPV4.5 (developed by Dr John Dempster, University of Strathclyde, UK). The data were filtered at 1 kHz and analysed offline using Clampfit 10.2 software or WinWCPV4.5 and Origin 9.1 software (OriginLab, Northampton, MA). The bath solution consisted of SBS and the pipette solution (intracellular solution) contained 145‐mM CsCl, 2‐mM MgCl_2_, 10‐mM HEPES, 1‐mM EGTA (free acid), 5‐mM ATP (sodium salt), and 0.1‐mM NaGTP, titrated to pH 7.2 with CsOH. All solutions were filtered using a 0.2 μm filter (Sartorius, UK). Comparisons of peak current sizes mediated by EA‐activated TRPC1/4/5 channels in whole cells and excised outside‐out patches are provided in Figure S12.

### 2.6 Data and statistical analysis

The data and statistical analysis comply with the recommendations of the *British Journal of Pharmacology* on experimental design and analysis in pharmacology (Curtis et al., 2018). All data are presented as means ± SEM and *P* < .05 considered statistically significant after ANOVA (Friedman test and Dunn's multiple comparison test) for multiple groups (post hoc test was only applied when ANOVA gave *P* < .05). Data were analysed using GraphPad Prism. Representative data are presented as raw data. Where required for the construction of concentration–response curves and statistical analysis, data were normalized to the appropriate control responses as detailed in the figures. Unless indicated otherwise, data were obtained from 5 independent experiments. Each independent experiment consisted of six technical replicates (i.e., six wells of a 96‐well plate for each Flex Station assay), unless stated otherwise. No blinding or randomization was undertaken. Compound additions in calcium recording experiments were performed using automated liquid handling by the FlexStation according to predefined plate layouts and appropriate controls were included on every plate. Concentration–response curves were fitted in GraphPad Prism as using a four parameter curve fit, using raw data for EC_50_ calculations and normalized responses for IC_50_ calculations. The amplitudes of Ca^2+^ responses for different channels were measured at time points indicated in the corresponding figure legends.

### 2.7 Materials

AM237 was synthesized by modification of published procedures (Chenard & Gallaschun, 2014; Rubaiy, Ludlow, Henrot, et al., 2017) and purified to homogeneity according to ^1^H NMR, ^13^C NMR, ^19^F NMR, and HPLC analysis (see Figures S1–S5). Pico145 was prepared according to previously reported procedures (Rubaiy, Ludlow, Henrot, et al., 2017). EA was obtained from PhytoLab (Vestenbergsgreuth, Germany). AM237, Pico145, and EA were made up as 10‐mM stocks in 100% DMSO, aliquots of which were stored at −20°C (AM237 and Pico145) or −80°C (EA). Further dilutions of compounds were made in DMSO and these were dissolved 1:1,000 in compound buffer (SBS + 0.01% pluronic acid) before being added to cells. Fura‐2 AM (Invitrogen UK) was dissolved at 1 mM in DMSO. http://www.guidetopharmacology.org/GRAC/LigandDisplayForward?ligandId=2436 and http://www.guidetopharmacology.org/GRAC/LigandDisplayForward?ligandId=2500 (Sigma‐Aldrich, Gillingham, UK) were made up in DMSO and stored at −80°C. Sphingosine‐1‐phosphate (S1P; Bio‐techne Ltd., Abingdon, UK) was dissolved in MeOH and stored at −80°C. http://www.guidetopharmacology.org/GRAC/LigandDisplayForward?ligandId=4357 was obtained from Sigma‐Aldrich (Gillingham, UK).

### 2.8 Nomenclature of targets and ligands

Key protein targets and ligands in this article are hyperlinked to corresponding entries in http://www.guidetopharmacology.org, the common portal for data from the IUPHAR/BPS Guide to PHARMACOLOGY (Harding et al., 2018), and are permanently archived in the Concise Guide to PHARMACOLOGY 2017/18 (Alexander et al., 2017).

## 3 RESULTS

### 3.1 AM237 activates homomeric TRPC5:C5 channels

**Figure 2:**
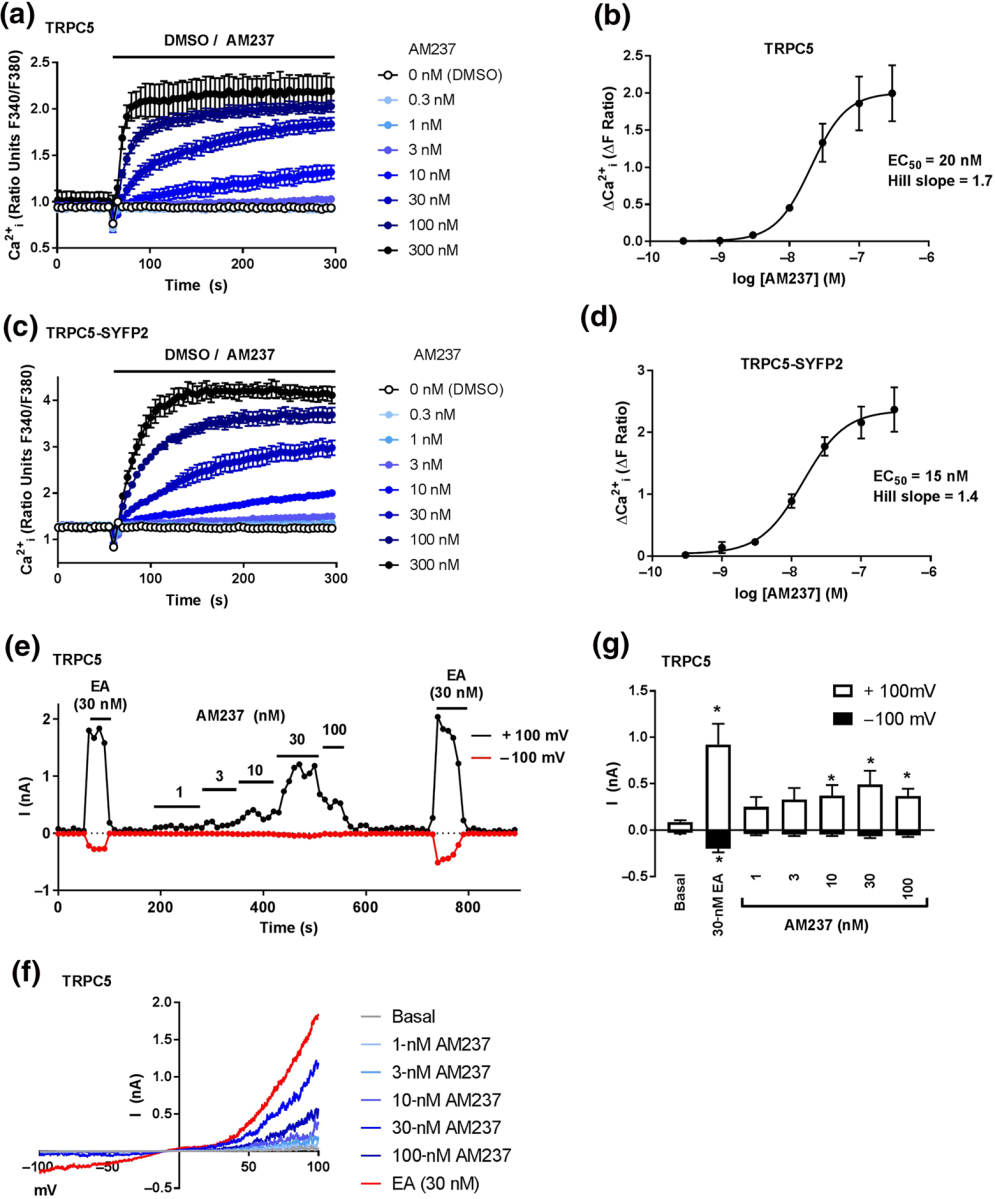
AM237 activates TRPC5:C5 channels. (a,c) Representative [Ca^2+^]_i_ measurements from single 96‐well plates (*N* = 6) showing an increase in [Ca^2+^]_i_ in response to 0.3‐ to 300‐nM AM237 in (Tet+) HEK T‐REx cells expressing hTRPC5 (a) or hTRPC5‐SYFP2 (c). (b,d) Concentration–response data for experiments in (a) and (c), respectively, showing mean responses ± SEM (*n*/*N* = 5/30). Responses were calculated at 250–295 s compared to [Ca^2+^]_i_ at baseline (0–55 s). (e) Typical outside‐out patch‐clamp data from a hTRPC5‐expressing HEK (Tet+) T‐REx cell showing current sampled at −100 and +100 mV during ramp changes in voltage (all agents were bath applied). (f) Representative current–voltage relationship (I–Vs) from experiments of the type illustrated in (e). (g) Mean responses ± SEM (*n* = 7 independent recordings) as shown in (e) for +100 mV and −100 mV. *P<0.05, significantly different from basal, one‐way ANOVA with Friedman's post hoc test

To test the activation of TRPC5:C5 channels by AM237, HEK T‐REx cells overexpressing TRPC5 or TRPC5‐SYFP2 were exposed to various concentrations of AM237 while monitoring [Ca^2+^]_i_ through ratiometric fluorescence measurements. Concentrations of 3 nM and above of AM237 led to a sustained increase in [Ca^2+^]_i_, which reached a maximal level around 40 s after exposure with 300‐nM AM237 (Figure 2a,c). Maximum responses were observed at 100–300 nM for TRPC5 and TRPC5‐SYFP2 channels. AM237 activated TRPC5 and TRPC5‐SYFP2 channels with similar EC_50_ values of 20 and 15 nM, respectively (Figure 2b,d). AM237 did not increase [Ca^2+^]_i_ in the absence of extracellular Ca^2+^ or in wild‐type HEK 293 cells (Figures S6 and S7), suggesting that the AM237‐induced Ca^2+^ response is mediated by TRPC5 channels expressed on the plasma membrane. Activation of TRPC5 channels by AM237 was also tested in electrophysiological (patch‐clamp) recordings. AM237 activated TRPC5 currents in excised outside‐out membrane patches of HEK T‐REx cells expressing TRPC5 (Figure 2e–g) and activation was reversible upon wash out, suggesting a relatively direct and membrane‐delimited effect. Amplitudes of responses to AM237 were lower than those to EA and were voltage dependent (up to 25% for inward currents and up to 45% for outward currents when compared to the responses induced by 30‐nM EA). Maximum responses were seen at 30‐nM AM237, with apparently lower responses at 100‐nM. The characteristic chair‐shape of the current–voltage plots was consistent with activation of homomeric TRPC5:C5 channels (Figure 2f; Ludlow et al., 2017; Rubaiy, Ludlow, Henrot, et al., 2017). These data suggest that AM237 is a potent activator of homomeric TRPC5:C5 channels.

### 3.2 AM237 suppresses EA‐mediated TRPC5:C5 channel activation

**Figure 3:**
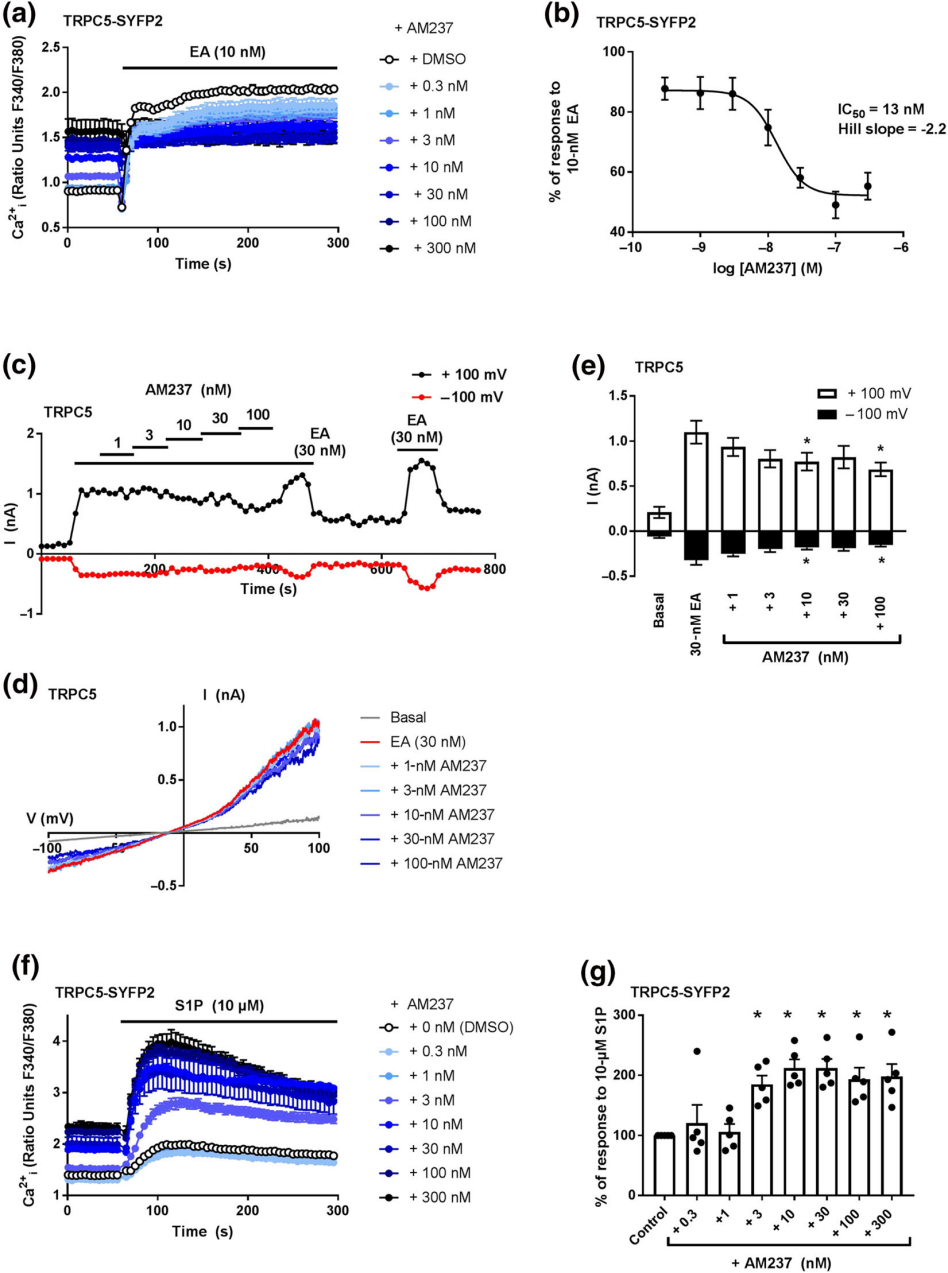
AM237 suppresses TRPC5:C5 activation by EA and potentiates TRPC5:C5 activation by S1P. (a) Representative [Ca^2+^]_i_ measurements from a single 96‐well plate (*N* = 6) showing an increase in basal [Ca^2+^]_i_ upon pre‐incubation of hTRPC5‐SYFP2 expressing (Tet+) HEK T‐REx cells with 0.3‐ to 300‐nM AM237, and the responses upon subsequent addition of 10‐nM EA. (b) Concentration–response data for experiments in (a), showing the suppression of the EA response by AM237. Data show mean responses ± SEM (*n*/*N* = 5/30). % response was calculated using response at 250–295 s compared to [Ca^2+^]_i_ at baseline (0–55 s). (c) Typical outside‐out patch‐clamp data from a hTRPC5‐expressing HEK (Tet+) T‐REx cell showing current sampled at −100 and +100 mV during ramp changes in voltage. Currents invoked by 30‐nM EA were suppressed by AM237 (all agents were bath applied). (d) Representative current–voltage relationship (I–Vs) from experiments of the type illustrated in (c). (e) Mean responses ± SEM (*n* = 7 independent recordings) as illustrated in (c) for +100 and −100 mV. *P<0.05, significantly different from 30 nM EA, one‐way ANOVA with Friedman's post hoc test. (f) Representative [Ca^2+^]_i_ measurements from a single 96‐well plate (*N* = 6) showing an increase in basal [Ca^2+^]_i_ upon pre‐incubation of hTRPC5‐SYFP2 expressing (Tet+) HEK T‐REx cells with 0.3‐ to 300‐nM AM237 and the response upon subsequent addition of 10‐μM S1P. (g) Mean responses ± SEM (*n*/*N* = 5/30) as illustrated in (f). Responses were calculated at 100–150 s compared to [Ca^2+^]_i_ at baseline (0–55 s). *P<0.05, significantly different from control, one‐way ANOVA with Dunnett's post hoc test

To assess the effect of AM237 on EA‐mediated TRPC5:C5 activation, HEK T‐REx cells overexpressing TRPC5‐SYFP2 were incubated with various concentrations of AM237 for 30 min before addition of EA. [Ca^2+^]_i_ was monitored through ratiometric fluorescence measurements. Consistent with the activating effects described above, AM237 increased basal [Ca^2+^]_i_ before addition of EA (Figure 3a). In the absence of AM237, addition of EA led to a rapid and sustained increase in [Ca^2+^]_i_. However, AM237 concentration‐dependently suppressed further activation of Ca^2+^ influx mediated by EA, with the largest effect at 100‐ to 300‐nM AM237 and with a relative IC_50_ value of 13 nM (Figure 3b). This effect of AM237 on the EA‐mediated responses of TRPC5:C5 channels was confirmed by patch‐clamp electrophysiology experiments, in which application of AM237 partially suppressed inward and outward currents induced by EA in outside‐out membrane patches (Figure 3c–e). The characteristic chair‐shape of the current–voltage plots was consistent with activation of homomeric TRPC5:C5 channels (Figure 3d; Ludlow et al., 2017; Rubaiy, Ludlow, Henrot, et al., 2017). Incubation with AM237 did not lead to reduced HEK T‐REx cell viability (Figure S8). These data suggest that AM237 acts as a partial agonist of TRPC5:C5 channels, compared with EA.

### 3.3 AM237 potentiates S1P‐mediated TRPC5:C5 channel activation

To assess the effect of AM237 on activation of TRPC5:C5 activation by agonists other than EA, HEK T‐REx cells overexpressing TRPC5‐SYFP2 were incubated with increasing concentration of AM237 for 30 min before addition of http://www.guidetopharmacology.org/GRAC/LigandDisplayForward?ligandId=911. S1P has been shown to activate TRPC5 channels via G‐protein signalling and does not mediate intracellular calcium release in HEK T‐REx cells (Xu et al., 2006). [Ca^2+^]_i_ was monitored through ratiometric fluorescence measurements. As expected, AM237 increased basal [Ca^2+]^_i_ before the addition of S1P (Figure 3f). Addition of S1P led to a rapid increase in [Ca^2+]^_i_, which reached a peak at around 60 s after application and then slowly declined. Addition of 0.3‐ to 1‐nM AM237 had no significant effect on the S1P‐mediated response. However, higher concentrations of AM237 (3–300 nM) significantly increased the activation of TRPC5:C5 channels by S1P (Figure 3g). In order to eliminate potential secondary effects resulting from prolonged incubation with AM237 before S1P administration, acute calcium responses, upon simultaneous addition of S1P and AM237 (0.3–300 nM), were measured as well. This produced similar concentration‐dependent increases of the responses to S1P, by AM237 (Figure S9). These data suggest that AM237 potentiates responses of TRPC5:C5 channels to S1P.

### 3.4 AM237 does not activate heteromeric TRPC1/5 channels but inhibits their EA‐mediated activation

**Figure 4:**
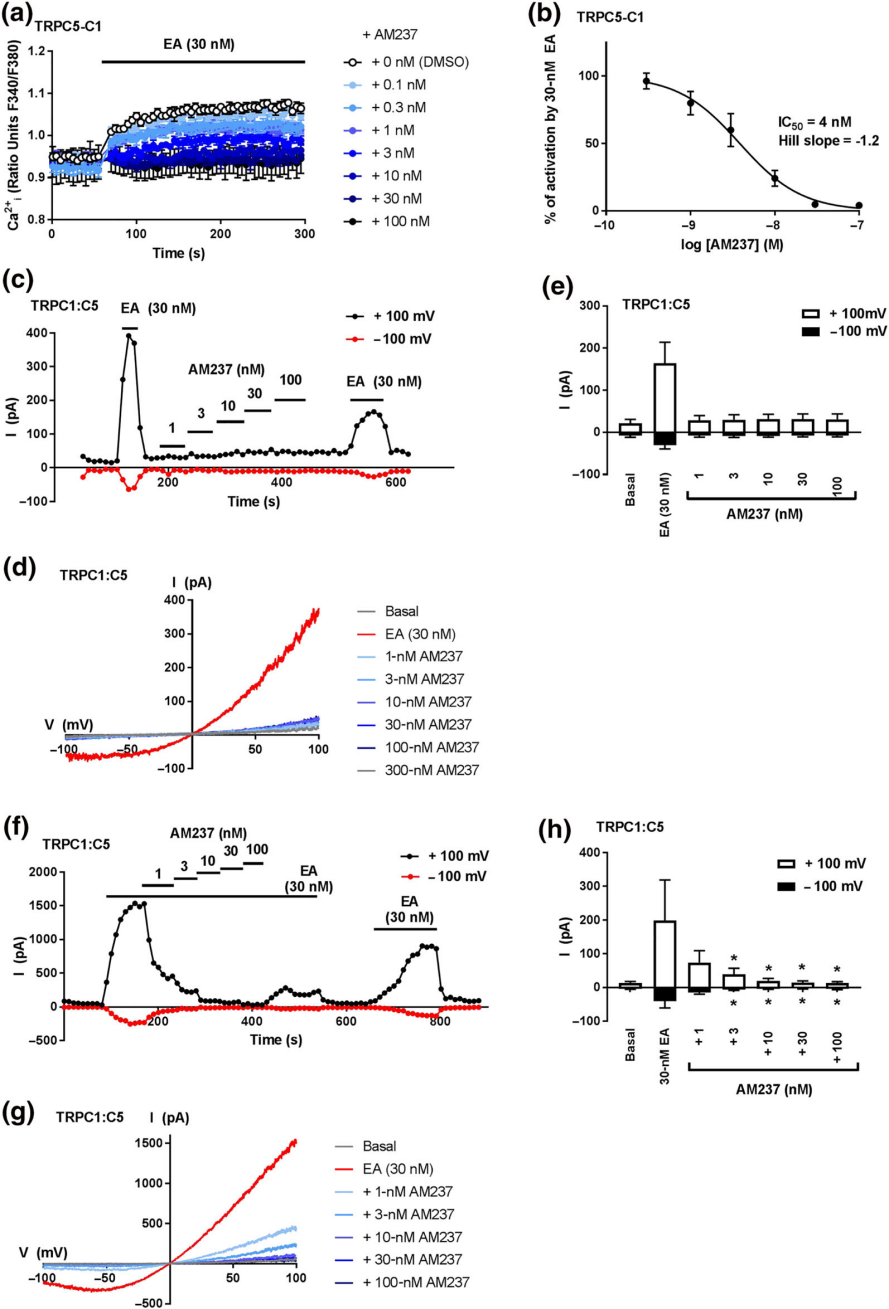
AM237 inhibits EA‐mediated activation of heteromeric TRPC1/5 channels. (a) Representative [Ca^2+^]_i_ measurements from a single 96‐well plate (*N* = 6) showing inhibition of EA‐mediated [Ca^2+^]_i_ responses by 0.1‐ to 100‐nM AM237 in (Tet+) HEK T‐REx cells expressing hTRPC5–C1. For these channels, no increases in [Ca^2+^]_i_ were seen upon pre‐incubation with AM237. (b) Concentration–response data for experiments in (a), showing mean responses ± SEM (*n*/*N* = 5/30). Responses were calculated at 250–295 s compared to [Ca^2+^]_i_ at baseline (0–55 s). (c,f) Example outside‐out patch‐clamp data HEK 293 cells transiently co‐expressing hTRPC1 and hTRPC5 showing current sampled at −100 and +100 mV during ramp changes in voltages to test activation by AM237 (c) and inhibition of EA‐induced currents by AM237 (f) (all agents were bath‐applied). (d,g) Representative current–voltage relationship (I–Vs) from experiments of the type illustrated in (c,f). (e,h) Mean responses ± SEM (*n* = 5 independent recordings) as illustrated in (c,f) for +100 and −100 mV. In (e), *P<0.05, significantly different from basal, one‐way ANOVA with Friedman's post hoc test; in (h), *P<0.05, significantly different from 30‐nM EA, one‐way ANOVA with Friedman's post hoc test. Overall, AM237 did not induce TRPC1:C5 currents (c–e) but currents invoked by 30‐nM EA were inhibited by AM237 (f–h)

To assess the activity of AM237 on heteromeric TRPC1/5 channels, HEK T‐REx cells overexpressing the TRPC5–C1 concatemer were incubated with various concentrations of AM237 before addition of EA. In contrast to its effects in cells overexpressing TRPC5 and TRPC5‐SYFP2, AM237 did not raise basal [Ca^2+^]_i_ in cells expressing TRPC5–C1 channels (Figure 4a), which is consistent with the lack of activation of TRPC5–C1 channels upon acute AM237 administration (Figure S10A–B). However, AM237 pretreatment concentration‐dependently inhibited the EA‐mediated increase in [Ca^2+^]_i_ in these cells with an IC_50_ of 4 nM (Figure 4a,b). These effects of AM237 were confirmed by patch‐clamp recordings on excised outside‐out membrane patches of cells transiently co‐expressing TRPC1 and TRPC5. In these experiments, AM237 did not activate TRPC1:C5 currents (Figure 4c–e) but inhibited currents induced by EA in a concentration‐dependent manner, with full inhibition of both inward and outward currents observed at 3‐ to 100‐nM AM237 (Figure 4f–h). The characteristic ladle‐shape of the current–voltage plots was consistent with activation of heteromeric TRPC1:C5 channels (Figure 4d,g; Ludlow et al., 2017; Rubaiy, Ludlow, Henrot, et al., 2017). These data suggest that AM237 is a potent inhibitor of heteromeric TRPC1:5 channels.

### 3.5 AM237 does not activate TRPC4 channels but inhibits their EA‐mediated activation

**Figure 5:**
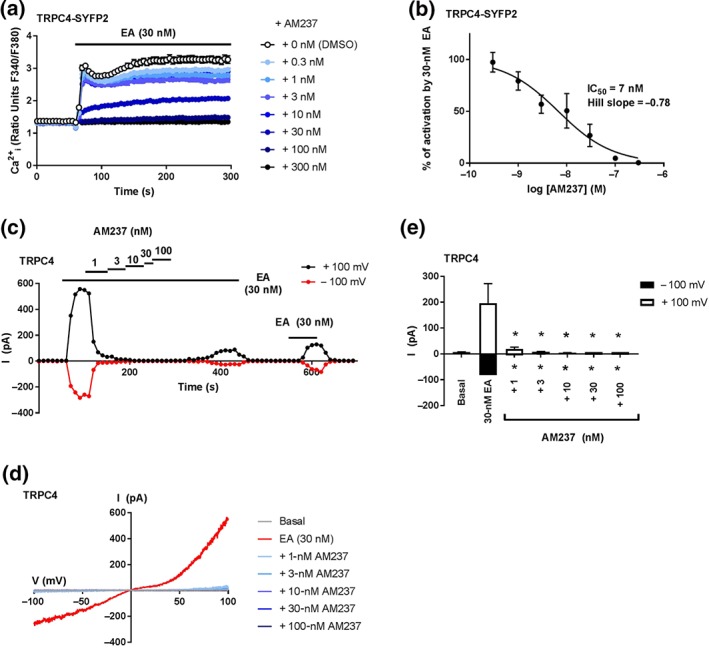
AM237 inhibits EA‐mediated activation of TRPC4 channels. (a) Representative [Ca^2+^]_i_ measurements from a single 96‐well plate (*N* = 6) showing inhibition of EA‐mediated [Ca^2+^]_i_ responses by 0.3‐ to 300‐nM AM237 in (Tet+) HEK T‐REx cells expressing hTRPC4‐SYFP2. For these channels, no increases in [Ca^2+^]_i_ were seen upon pre‐incubation with AM237. (b) Concentration–response data for experiments in (a), showing mean responses ± SEM (*n*/*N* = 5/30). Responses were calculated at 250–295 s compared to [Ca^2+^]_i_ at baseline (0–55 s). (c) Example outside‐out patch‐clamp data of HEK 293 cells transiently expressing hTRPC4 showing current sampled at −100 and +100 mV during ramp changes in voltage to test inhibition of EA‐induced currents by AM237 (all agents were bath applied). (d) Representative current–voltage relationship (I–Vs) from experiments of the type illustrated in (c). (e) Mean responses ± SEM (*n* = 6–7 independent recordings) as illustrated in (c) for −100 and +100 mV. Currents evoked by 30 nM EA were inhibited by AM237. *P<0.05, significantly different from 30‐nM EA, one‐way ANOVA with Friedman's post hoc test

**Figure 6:**
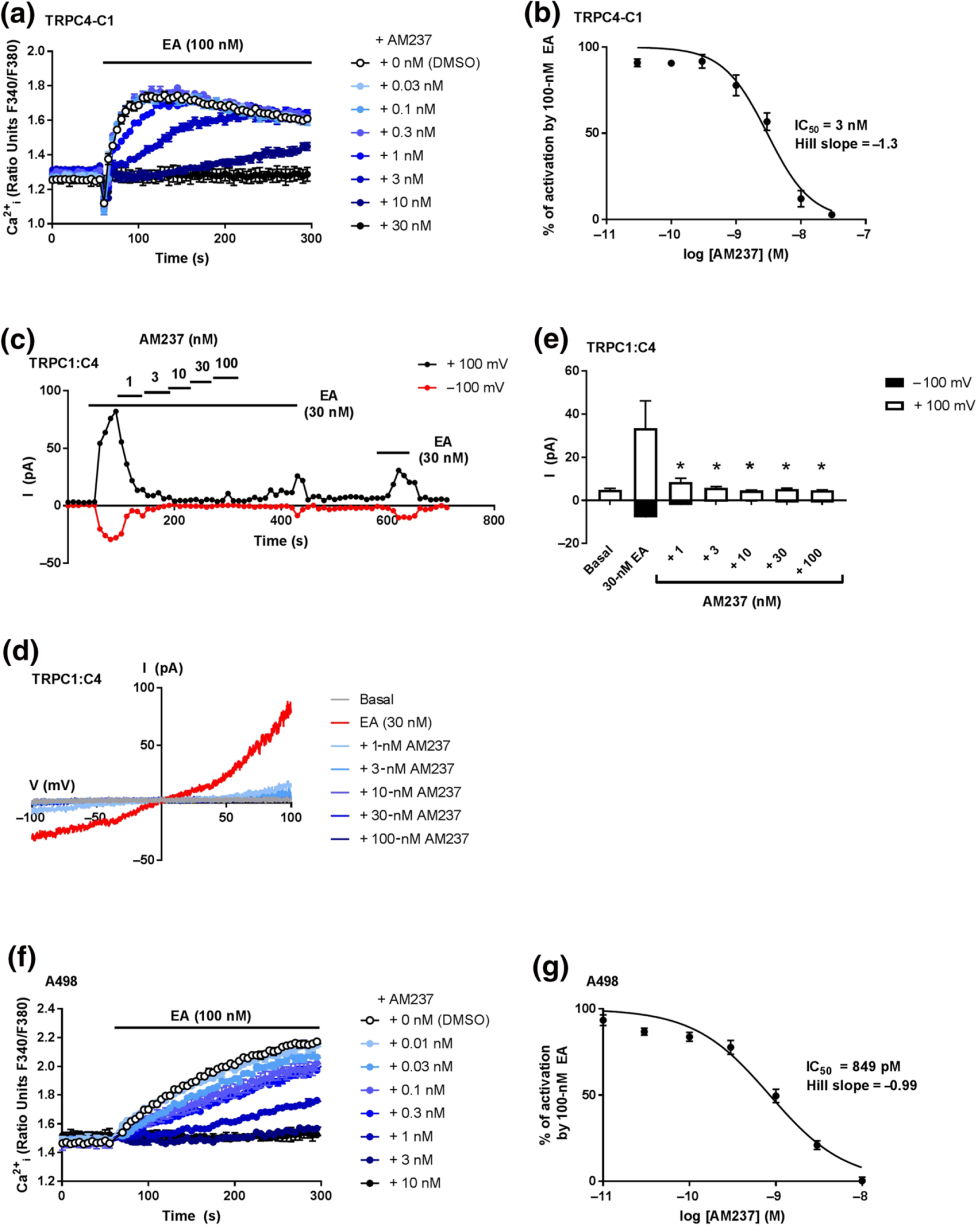
AM237 inhibits EA‐mediated activation of overexpressed and native TRPC1:C4 channels. (a) Representative [Ca^2+^]_i_ measurements from a single 96‐well plate (*N* = 6) showing inhibition of EA‐mediated [Ca^2+^]_i_ responses by 0.03‐ to 30‐nM AM237 in (Tet+) HEK T‐REx cells expressing hTRPC4‐C1. For these channels, no increases in [Ca^2+^]_i_ were seen upon pre‐incubation with AM237. (b) Concentration–response data for experiments in (a), showing mean responses ± SEM (*n*/*N* = 5/30). Responses were calculated at 150–200 s compared to [Ca^2+^]_i_ at baseline (0–55 s). (c) Example outside‐out patch‐clamp data of HEK 293 cells transiently co‐expressing hTRPC4 and hTRPC1 showing current sampled at −100 and +100 mV during ramp changes in voltage to test inhibition of EA‐induced currents by AM237 (all agents were bath applied). (d) Representative current–voltage relationship (I–Vs) from experiments of the type illustrated in (c). (e) Mean responses ± SEM (*n* = 5 independent recordings) as illustrated in (c) for −100 and +100 mV. Currents evoked by 30‐nM EA were inhibited by AM237. *P < 0.05, significantly different from 30 nM EA, one‐way ANOVA with Friedman's post hoc test. (f) Representative [Ca^2+^]_i_ measurements from a single 96‐well plate (*N* = 6) showing inhibition of EA‐mediated [Ca^2+^]_i_ responses by 0.01‐ to 10‐nM AM237 in A498 cells, which natively express TRPC1:C4 channels. For these cells, no increases in [Ca^2+^]_i_ were seen upon pre‐incubation with AM237. (g) Concentration–response data for experiments in (f), showing mean responses ± SEM (*n*/*N* = 5/30). Responses were calculated at 250–295 s compared to [Ca^2+^]_i_ at baseline (0–55 s)

To assess the activity of AM237 on TRPC4:C4 (Figure 5) and TRPC1:C4 (Figure 6) channels, HEK T‐REx cells overexpressing TRPC4‐SYFP2 or concatemeric TRPC4–C1 were incubated with various concentrations of AM237 before addition of EA, and [Ca^2+^]_i_ was monitored. For both channels, AM237 pretreatment did not affect basal [Ca^2+^]_i_ (Figures 5a and 6a), which is consistent with the lack of channel activation upon acute AM237 administration (Figure S10C–F). However, AM237 concentration dependently inhibited the EA‐mediated increase in [Ca^2+^]_i_ with IC_50_ values of 7 nM (TRPC4‐SYFP2; Figure 5a,b) and 3 nM (TRPC4–C1; Figure 6a,b) respectively. The effects of AM237 on EA‐mediated activation of TRPC4 and TRPC4:C1 were confirmed by patch‐clamp recordings on excised outside‐out membrane patches of cells transiently expressing TRPC4 or co‐expressing TRPC1 and TRPC4. In these experiments, 1‐ to 100‐nM AM237 inhibited currents induced by EA in both TRPC4 (Figure 5c–e) and TRPC4:C1 channels (Figure 6c–e), with full inhibition observed at 3‐ to 100‐nM AM237. In order to test these effects of AM237 on endogenous TRPC1:C4 channels, A498 renal carcinoma cells, which endogenously express TRPC1:C4 channels (Akbulut et al., 2015; Carson et al., 2015; Ludlow et al., 2017), were pretreated with various concentrations of AM237 before the addition of EA. Consistent with the absence of channel activation upon acute AM237 administration (Figure S10G,H), pretreatment with AM237 had no effect on basal [Ca^2+^]_i_, but concentration dependently inhibited the EA‐mediated increase in [Ca^2+^]_i_ with an IC_50_ of 849 pM (Figure 6f,g). These data suggest that AM237 is a potent inhibitor of homomeric and heteromeric TRPC4 channels.

### 3.6 AM237 does not activate or inhibit TRPC3, TRPC6, TRPV4, or TRPM2 channels

The selectivity of AM237 against other TRP channels was tested by [Ca^2+^]_i_ measurements. At 300‐nM AM237 (a concentration that gives maximum effect in all [Ca^2+^]_i_ measurements with TRPC1/4/5 channels), no activation or inhibition was seen in cells expressing TRPC3, TRPC6, TRPV4, or TRPM2 channels (Figure S11). These data suggest that AM237 has selectivity for TRPC1/4/5 channels.

### 3.7 Pico145 is a competitive antagonist of AM237‐mediated TRPC5:C5 activation

**Figure 7:**
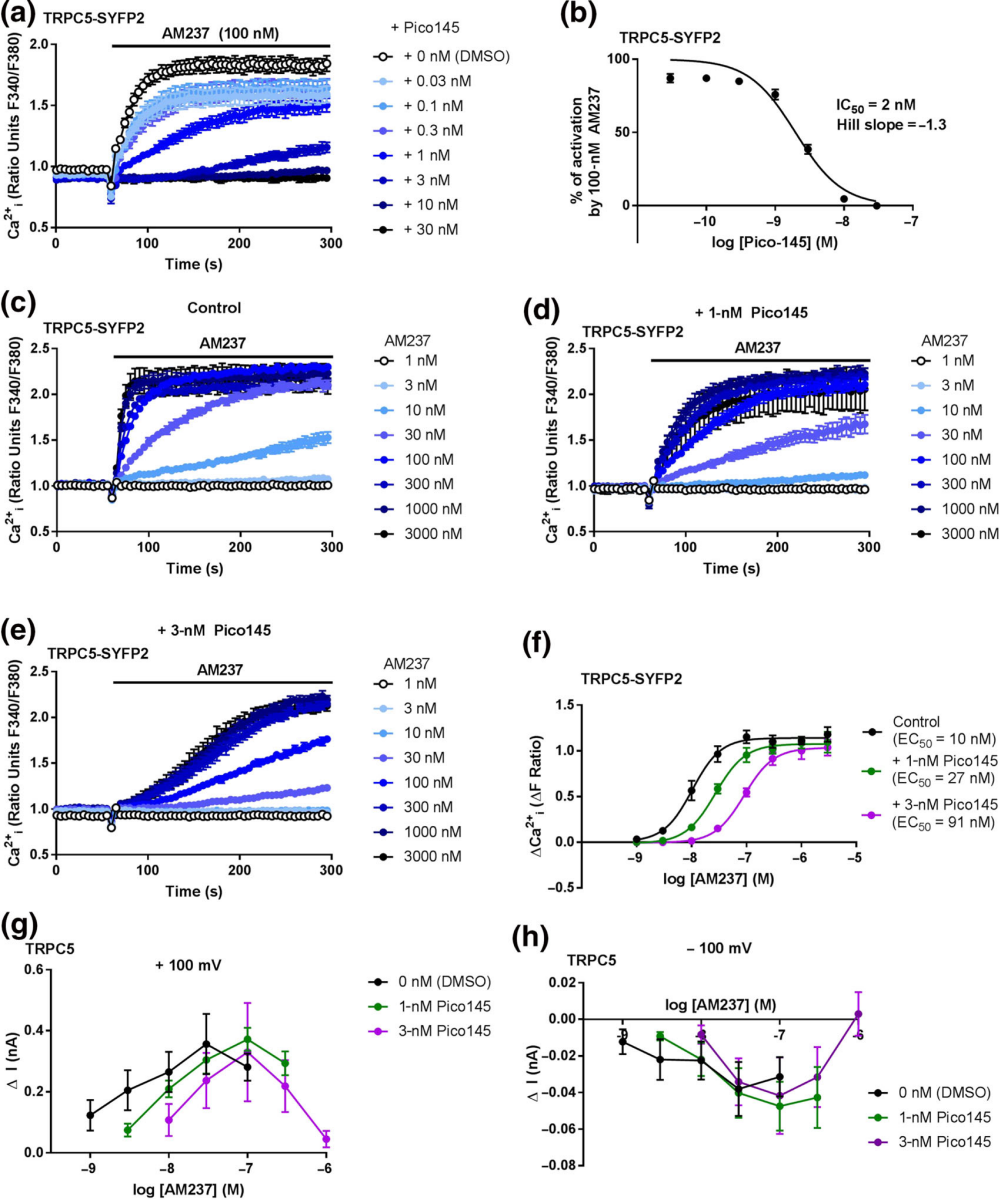
Pico145 inhibits activation of TRPC5:C5 by AM237. (a) Representative [Ca^2+^]_i_ measurements from a single 96‐well plate (*N* = 6) showing inhibition by 0.03‐ to 30‐nM Pico145 of the [Ca^2+^]_i_ response evoked by 100‐nM AM237 in hTRPC5‐SYFP2 expressing (Tet+) HEK T‐REx cells. (b) Concentration–response data for experiments in (a), showing mean ± SEM (*n*/*N* = 5/30). Responses were calculated at 250–295 s compared to [Ca^2+^]_i_ at baseline (0–55 s). (c–e) Representative [Ca^2+^]_i_ measurements from single 96‐well plates (*N* = 4) showing the response of 1‐ to 3,000‐nM AM237 in hTRPC5‐SYFP2 expressing (Tet+) HEK T‐REx cells pretreated with DMSO (vehicle) (c), 1‐nM Pico145 (d), or 3‐nM Pico145 (e). Note that higher Pico145 concentrations resulted in slower increases of [Ca^2+^]_i_. (f) Concentration–response data showing mean responses ± SEM (*n*/*N* = 5/20) for experiments in (c–e). The parallel rightwards shift of the EC_50_ curves at increasing [Pico145] suggest that Pico145 is a competitive antagonist of AM237 in the context of TRPC5:C5. (g,h) Dose–response data for outside‐out patch‐clamp recordings from hTRPC5‐expressing HEK (Tet+) T‐REx cells, showing mean responses ± SEM (*n* = 5 independent experiments) with currents sampled at −100 mV (g) and +100 mV (h). After the response to EA (30 nM) was confirmed, excised outside‐out patches were pre‐incubated with DMSO (vehicle), 1‐nM Pico145 or 3‐nM Pico145, and then treated with different concentrations of AM237. The rightwards shift of dose–response traces suggest that Pico145 inhibits AM237‐evoked currents mediated by TRPC5:C5 channels. At higher concentrations, inhibitory effects of AM237 were seen in these recordings

To assess if the xanthine Pico145 could inhibit TRPC5:C5 channel activation by AM237, HEK T‐REx cells overexpressing TRPC5‐SYFP2 were incubated with either DMSO (vehicle) or Pico145 (0.03–30 nM) for 30 min before addition of 100‐nM AM237 and [Ca^2+^]_i_ was monitored. Pico145 concentration dependently inhibited the response of TRPC5:C5 to AM237, with maximal inhibition at 10 nM of Pico145 and an IC_50_ of 2 nM (Figure 7a,b). These values are similar to those observed for inhibition by Pico145 of EA‐mediated TRPC5:C5 activation (IC_50_ = 1 nM when 10 nM EA was used; Rubaiy, Ludlow, Henrot, et al., 2017). To test if Pico145 and AM237 are competitive TRPC5:C5 modulators, concentration‐dependent responses of TRPC5:C5 to AM237 were measured in the absence of Pico145 and in the presence of two different concentrations of Pico145 (1 and 3 nM). Pre‐incubation with Pico145 slowed down the increase in [Ca^2+^]_i_ and some of the AM237 responses may not have reached steady state (Figure 7c–e). However, the inhibition by Pico145 was surmountable and increasing concentrations of AM237 caused parallel rightward shifts in the AM237 concentration–response curve (Figure 7f). These findings were confirmed by patch‐clamp recordings on excised outside‐out membrane patches of cells expressing TRPC5. In the absence of Pico145, AM237 (1–30 nM) concentration dependently increased currents recorded at both +100 and −100 mV, with a decrease in the response at 100‐nM AM237 (Figure 7g,h). In the presence of 1‐ and 3‐nM Pico145, increasing concentrations of AM237 were required to activate the same current, with a peak current being recorded at 100 nM and decreasing at 300 nM and 1 μM (Figure 7g,h). These data suggest that Pico145 is a competitive antagonist of AM237 in the context of homomeric TRPC5:C5 channels.

## 4 DISCUSSION AND CONCLUSIONS

This study describes the effects of a close analogue of the TRPC1/4/5 inhibitor Pico145 (Rubaiy, Ludlow, Henrot, et al., 2017) on TRPC1/4/5 channels. The xanthine AM237 differs from Pico145 by only a single chloride substituent on one of its aryl rings. However, whereas Pico145 is a potent TRPC5:C5 inhibitor (IC_50_ ~1 nM), AM237 is a potent TRPC5:C5 activator (EC_50_ = 10–20 nM; Figures 2 and 7) that can also potentiate responses of TRPC5:C5 channels to S1P (Figure 3f–h). In outside‐out patch‐clamp experiments, the size of AM237‐evoked TRPC5:C5 currents was voltage dependent, at 25–45% of currents evoked by EA, the most efficacious TRPC1/4/5 activator known to date (Figure 2e–g). However, in contrast to EA, AM237 is a highly selective activator of homomeric TRPC5:C5 channels, as it does not activate homomeric TRPC4:C4 channels or heteromeric TRPC1/5 and TRPC1/4 channels. Instead, and similar to Pico145, it strongly inhibits the response of these other TRPC1/4/5 channels to EA (IC_50_ values ranging from 3 to 7 nM; Figures 4 and 5). The additional observation that AM237 suppresses EA‐induced TRPC5:C5 activity suggests that EA and AM237 compete for binding to their cellular targets, potentially because the compounds have shared or overlapping binding sites, although allosteric regulation of binding sites cannot be excluded. These data, combined with earlier reports that Pico145 itself has both inhibiting and potentiating effects, depending on the presence of other activators (Rubaiy, Ludlow, Henrot, et al., 2017), suggest that xanthine‐based compounds can stabilize open or closed TRPC5 channel states, depending on subunit composition and xanthine substituent pattern.

The strong dependence of the functional effect of xanthine derivatives on TRPC1/4/5 channels on channel composition and xanthine structure is consistent with the hypothesis that xanthines bind directly to the channels, through a mechanism different from block of the ion pore (Rubaiy, Ludlow, Henrot, et al., 2017). This is reinforced by the finding that AM237, like Pico145, modulates TRPC1/4/5 channels in excised outside‐out membrane patches in a reversible manner (Figures 2, 3, 4, 5, 6). It should be noted that the patch‐clamp data suggest that, under these conditions, the EC_50_ and IC_50_ values of AM237 are lower than in [Ca^2+^]_i_ measurements, which is consistent with effects reported for Pico145 (Rubaiy, Ludlow, Henrot, et al., 2017). In addition, in some recordings, the recovery of EA‐induced currents was incomplete (Figures 4c,f, 5c, and 6c), which suggests a possible contribution of TRPC1/4/5 channel desensitization to the inhibitory effects of AM237.

The finding that Pico145 is a competitive antagonist of AM237 at TRPC5:C5 channels (Figure 7), in combination with the high structural similarity of these two compounds, suggests that AM237 and Pico145 have a common binding site. It is possible that multiple xanthines bind to each tetrameric TRPC1/4/5 channel; Hill slopes >1 found in most concentration–response curves (and especially for TRPC5‐containing channels) may indeed indicate cooperative effects. However, the exact stoichiometry of binding of xanthines to TRPC1/4/5 channels cannot be determined from these data, because not every binding event may have the same effect on stabilization of different channel states. In addition, differences between TRPC5 [Ca^2+^]_i_ measurements and TRPC5 patch‐clamp recordings were observed at higher concentrations of AM237 (e.g., Figure 7g,h vs. Figure 7f). A potential explanation for this is the difference between experimental protocols, which include differences in cell preparation (highly confluent cells in recording buffer vs. excised membrane patches from sparse single cells that were strongly dialyzed on both sides of the membrane), compound application protocols (single concentrations in [Ca^2+^]_i_ measurements vs. accumulative application in patch recordings), ion fluxes measured (such as the significant monovalent cation fluxes that contribute to currents in patch recordings are not detected in [Ca^2+^]_i_ measurements), and timeframes of experiments (slow accumulation of [Ca^2+^]_i_ vs. rapid current recordings).

Only few TRPC1/4/5 modulators are known to clearly distinguish between different TRPC1/4/5 tetramers. Pico145 inhibits all TRPC1/4/5 channels but is more potent against heteromeric channels, and especially TRPC1/4 channels (Rubaiy, Ludlow, Henrot, et al., 2017). However, the range of potencies of Pico145 against different TRPC1/4/5 tetramers may depend on channel activation mode (Just et al., 2018; Rubaiy, Ludlow, Henrot, et al., 2017). The benzothiadiazine derivative BTD activates TRPC5:C5 (EC_50_ ~1 μM), TRPC1:C5, and TRPC4:C5 channels, but not TRPC4:C4 or TRPC1:C4 channels (Beckmann et al., 2017). The benzimidazole derivative http://www.guidetopharmacology.org/GRAC/LigandDisplayForward?ligandId=10290 was recently reported as a TRPC5 channel inhibitor that does not inhibit TRPC4 channels (Sharma, Pablo, Montesinos, Greka, & Hopkins, 2018; Zhou et al., 2017). However, effects on heteromeric TRPC1/4/5 channels were not reported for this compound (or its analogues). http://www.guidetopharmacology.org/GRAC/LigandDisplayForward?ligandId=4255 is a micromolar TRPC4/5 inhibitor with limited effects on hundreds of other proteins (Miller et al., 2011), but it is a relatively poor inhibitor of heteromeric TRPC1/4 and TRPC1/5 channels, at least when channels are activated with EA (Muraki et al., 2017; Rubaiy, Ludlow, Henrot, et al., 2017). To the best of our knowledge, AM237 is the first TRPC1/4/5 modulator with nanomolar potency that clearly distinguishes homomeric TRPC5:C5 channels from other TRPC1/4/5 tetramers. Therefore, AM237 may be a useful chemical tool to help understand the composition of TRPC1/4/5 channels and the biological roles of specific channels, in different cell types.

## AUTHOR CONTRIBUTIONS

A.M. performed synthesis and calcium measurements. C.C.B., I.B.P., and D.J.W. performed calcium measurements. K.M. performed patch‐clamp experiments. E.C.B. performed cell viability experiments. M.J.L. and E.C.B. developed TRPC4/5‐SYFP2 constructs. M.J.L., E.C.B., and C.C.B. generated stable cell lines. A.M., C.C.B., K.M., E.C.B., and R.S.B. analysed data. C.C.B., K.M., E.C.B., and R.S.B. made figures. M.P.B., S.L.W., and D.J.B. advised on experimental design and data interpretation and generated research funds. R.S.B. conceived the research, led the project, and generated research funds. C.C.B. and R.S.B. wrote the manuscript. All authors commented on the manuscript.

## CONFLICT OF INTEREST

The authors declare no conflicts of interest.

## DECLARATION OF TRANSPARENCY AND SCIENTIFIC RIGOUR

This Declaration acknowledges that this paper adheres to the principles for transparent reporting and scientific rigour of preclinical research as stated in the *BJP* guidelines for https://bpspubs.onlinelibrary.wiley.com/doi/full/10.1111/bph.14207, and as recommended by funding agencies, publishers and other organisations engaged with supporting research.

## Supporting information

Figure S1. Synthetic route to AM237
Figure S2. ^1^H NMR spectrum of AM237
Figure S3. ^13^C NMR spectrum of AM237
Figure S4. ^19^F NMR spectrum of AM237
Figure S5. HPLC chromatogram of AM237
Figure S6. AM237‐mediated increase of [Ca^2+^]i is dependent on the presence of extracellular calcium. A) [Ca^2+^]_i_ measurements from a single 96‐well plate (N = 6) showing that 0.3‐300 nM AM237 has no effect on [Ca^2+^]_i_ in TRPC5‐SYFP2 expressing (Tet+) HEK T‐REx cells in the absence of extracellular calcium. Both recording buffer and compounds were made up in Ca^2+^‐free SBS, where CaCl_2_ was replaced by 0.4 mM EGTA.
Figure S7. AM237‐mediated increase of [Ca^2+^]_i_ is dependent on the expression of TRPC5. A) [Ca^2+^]_i_ measurements from a single 96‐well plate (N = 6) showing that 0.3–300 nM AM237 has no effect on [Ca^2+^]_i_ in wild‐type HEK 293 cells. B) Example whole‐cell patch clamp data from a WT HEK 293 cell showing current sampled at ‐100 mV and +100 mV during ramp changes in voltage. AM237 and EA had no significant effect on currents recorded. C) Representative currentvoltage relationship (I‐Vs) from experiments of the type illustrated in (B). D) Mean response ± SEM (n = 5/6 independent recordings) as illustrated in (B) for +100 mV and ‐100 mV.
Figure S8. AM237 has no effect on cell viability of HEK T‐REx (Tet+) expressing TRPC5. A) Representative images from LIVE/DEAD® cell viability assay for HEK T‐REx (Tet +) cells expressing TRPC5 treated with AM237 (1 μM), DMSO (vehicle control) or methanol (positive control). Column 1, phase image of HEK T‐REx cells expressing TRPC5. Column 2, Calcein AM staining for live cells. Column 3, Ethidium homodimer‐1 (EthD‐1) staining for dead cells. Row 1, cells treated with 1 μM AM237. Row 2, cells treated with DMSO. Row 3, cells fixed with methanol prior to treatment with DMSO. B) Mean ± SEM from experiment in (A). Data from three independent experiments.
Figure S9. AM237 potentiates the S1P response of TRPC5:C5 channels. A) Representative [Ca^2+^]_i_ measurements from a single 96‐well plate (N = 6) showing an increase in [Ca^2+^]_i_ in response to 0.3‐300 nM AM237 in hTRPC5‐SYFP2 expressing (Tet+) HEK T‐REx cells. B) Representative [Ca^2+^]_i_ measurements from a single 96‐well plate (N = 6) showing an increase in [Ca^2+^]_i_ upon co‐application of 10 μM S1P and 0.3‐300 nM AM237 in hTRPC5‐SYFP2 expressing (Tet+) HEK T‐REx cells. C) Concentration‐response data for experiments in (F) and (G), showing mean responses ± SEM (n/N = 5/30). Responses were calculated at 250‐295 s compared to [Ca^2+^]_i_ at baseline (0‐55 s).
Figure S10. AM237 does not activate TRPC5:C1, TRPC4 and TRPC4:C1 channels A, C, E). Representative [Ca^2+^]_i_ measurements from single 96‐well plates (N = 6) showing an increase in [Ca^2+^]_i_ in response to 100 nM EA, and lack of activation with 3‐300 nM AM237 in (Tet+) HEK T‐REx cells expressing hTRPC5‐C1 (A), hTRPC4‐SYFP2 (C) and hTRPC4‐C1 (E). B, D, F) Mean ± SEM for experiments in (A), (C), and (E) respectively. Responses were calculated at 250‐295 s compared to basal [Ca^2+^]_i_ 0‐55 s (n/N = 3/18) G) Representative [Ca^2+^]_i_ measurements from a single 96‐well plate (N = 6) showing an increase in [Ca^2+^]_i_ in response to 100 nM EA, and lack of activation with 3‐300 nM AM237 in A498 cells. H) Mean ± SEM for experiments in (G). Responses were calculated at 250‐295 s compared to basal [Ca^2+^]_i_ 0‐55 s (n/N = 3/18)
Figure S11. AM237 has no effect on TRPC3, TRPC6, TRPV4 or TRPM2 channels. A, C) Representative [Ca^2+^]_i_ measurements from single 96‐well plates (N = 6) showing no effect of AM237 on activation of hTRPC3 (A) or hTRPC6 (C) by 100 μM OAG in WT HEK 293 cells transiently transfected with hTRPC3 (A) or hTRPC6 (C). The baselines before OAG application indicate that AM237 does not activate TRPC3 (A) or TRPC6 (C). Cells not expressing TRPC3 (A) or TRPC6 (C) do not respond to 100 μM OAG. B,D) Mean responses ± SEM (n/N = 3/18) for experiments shown in (A,C), for hTRPC3 (B) and hTRPC6 (D). Responses were calculated at 75‐ 95 s (TRPC3) and 80‐100 s (TRPC6) compared to basal [Ca^2+^]_i_ (0‐55 s). E) Representative [Ca^2+^]_i_ measurements from a single 96‐well plate (N = 6) showing no effect of AM237 on activation of TRPV4 by 5 μM 4α‐PDD in CHO cells stably expressing hTRPV4. The baselines before 4α‐PDD application indicate that AM237 does not activate TRPV4. F) Mean responses ± SEM (n/N = 3/18) for experiments shown in (E). Responses were calculated at 250‐295 s compared to basal [Ca^2+^]_i_ (0‐55 s). G) Representative [Ca^2+^]_i_ measurements from a single 96‐well plate (N = 6) showing no effect of AM237 on activation of TRPM2 by 1 mM H_2_O_2_ in (Tet+) HEK T‐REx cells expressing TRPM2. The baselines before H_2_O_2_ application indicate that AM237 does not activate TRPM2. Cells not expressing TRPM2 do not respond to H_2_O_2_. H) Mean responses ± SEM (n/N = 3/18) for experiments shown in (F). Responses were calculated at 550‐595 s compared to basal [Ca^2+^]_i_ (0‐55 s).
Figure S12. Differences in current amplitudes of TRPC5:C5, TRPC5:C1, TRPC4:C4 and TRPC4:C1 channels recorded by whole‐cell patch and outside‐out patch clamp electrophysiology. A) Mean ± SEM peak currents evoked by 100 nM EA in HEK 293 cells expressing either TRPC5, TPRC1 and TRPC5, TRPC4, or TRPC1 and TRPC4, measured by whole‐cell patch clamp electrophysiology, with current sampled at ‐80 mV (white bars) and +80 mV (black bars) (n = 4‐12). B) Mean ± SEM peak currents evoked by 100 nM EA in HEK 293 cells expressing either TRPC5, TPRC1 and TRPC5, TRPC4, or TRPC1 and TRPC4, measured in excised outside‐out membrane patches, with current sampled at ‐80 mV (white bars) and +80 mV (black bars) (n = 4‐8). Note the difference in scale of the y‐axes between (A) and (B).
