# Supplementary material for: Potent, selective, and subunit‐dependent activation of TRPC5 channels by a xanthine derivative
Source: Br J Pharmacol. 2019 Sep 6;176(20):3924–38. doi: 10.1111/bph.14791 (PMC6811774; doi:10.1111/bph.14791)
Supplement: Supplementary file 1 — Figure S1. Synthetic route to AM237 Figure S2. 1H NMR spectrum of AM237 Figure S3. 13C NMR spectrum of AM237 Figure S4. 19F NMR spectrum of AM237 Figure S5. HPLC chromatogram of AM237 Figure S6. AM237‐mediated increase of [Ca2+]i is dependent on the presence of extracellular calcium. A) [Ca2+]i measurements from a single 96‐well plate (N = 6) showing that 0.3‐300 nM AM237 has no effect on [Ca2+]i in TRPC5‐SYFP2 expressing (Tet+) HEK T‐REx cells in the absence of extracellular calcium. Both recording buffer and compounds were made up in Ca2+‐free SBS, where CaCl2 was replaced by 0.4 mM EGTA. Figure S7. AM237‐mediated increase of [Ca2+]i is dependent on the expression of TRPC5. A) [Ca2+]i measurements from a single 96‐well plate (N = 6) showing that 0.3–300 nM AM237 has no effect on [Ca2+]i in wild‐type HEK 293 cells. B) Example whole‐cell patch clamp data from a WT HEK 293 cell showing current sampled at ‐100 mV and +100 mV during ramp changes in voltage. AM237 and EA had no significant effect on currents recorded. C) Representative currentvoltage relationship (I‐Vs) from experiments of the type illustrated in (B). D) Mean response ± SEM (n = 5/6 independent recordings) as illustrated in (B) for +100 mV and ‐100 mV. Figure S8. AM237 has no effect on cell viability of HEK T‐REx (Tet+) expressing TRPC5. A) Representative images from LIVE/DEAD® cell viability assay for HEK T‐REx (Tet +) cells expressing TRPC5 treated with AM237 (1 μM), DMSO (vehicle control) or methanol (positive control). Column 1, phase image of HEK T‐REx cells expressing TRPC5. Column 2, Calcein AM staining for live cells. Column 3, Ethidium homodimer‐1 (EthD‐1) staining for dead cells. Row 1, cells treated with 1 μM AM237. Row 2, cells treated with DMSO. Row 3, cells fixed with methanol prior to treatment with DMSO. B) Mean ± SEM from experiment in (A). Data from three independent experiments. Figure S9. AM237 potentiates the S1P response of TRPC5:C5 channels. A) Representative [Ca2+]i measurements [file BPH-176-3924-s001.pdf]

Potent, selective and subunit-dependent activation of TRPC5 channels by a xanthine derivative

Aisling Minard[1,2,†], Claudia C. Bauer[2,†], Eulashini Chuntharpursat-Bon[2], Isabelle B. Pickles[1,2], David J. Wright[2], Melanie J. Ludlow[2], Matthew P. Burnham[3], Stuart L. Warriner[1], David J. Beech[2], Katsuhiko Muraki[4,*], Robin S. Bon[2,*]

[1]School of Chemistry, University of Leeds, Leeds LS2 9JT, UK. [2]Department of Discovery and Translational Science, Leeds Institute of Cardiovascular and Metabolic Medicine, University of Leeds, Leeds LS2 9JT, UK. [3]Discovery Sciences, R&B Biopharmaceuticals, AstraZeneca, Alderley Park, SK10 4TG, UK. [4]Laboratory of Cellular Pharmacology, School of Pharmacy, Aichi-Gakuin University, 1-100 Kusumoto, Chikusa, Nagoya 464-8650, Japan.

*Authors for correspondence: Dr Robin S. Bon, Leeds Institute of Cardiovascular and Metabolic Medicine, School of Medicine, LIGHT Building, Clarendon Way, University of Leeds, Leeds LS2 9JT, UK. Email r.bon@leeds.ac.uk; Prof. Katsuhiko Muraki, [4]Laboratory of Cellular Pharmacology, School of Pharmacy, Aichi-Gakuin University, 1-100 Kusumoto, Chikusa, Nagoya 464-8650, Japan. Email kmuraki@dpc.agu.ac.jp. [†]These authors contributed equally

Supporting information

Synthetic details

All chemical reagents were purchased from commercial suppliers and used without further purification. Anhydrous solvents and liquid reagents were purchased in Sure/Seal™ bottles. Flash column chromatography was carried out using silica (Merck Geduran silica gel, 35–70 µm particles). Thin layer chromatography was carried out on commercially available pre-coated aluminium plates (Merck silica 2 8 8 0 Kieselgel 60 F_{254}). Analytical HPLC was performed on an Agilent 1290 Infinity Series equipped with a UV detector and a Hyperclone C_{18} reverse phase column using MeCN/water (5→95%) containing 0.1% formic acid, at either 0.5 mL min^{-1} over a period of five minutes or 1.0 mL min^{-1} over a period of 30 minutes. High resolution electrospray (ESI+) mass spectrometry was performed on a Bruker MaXis Impact QTOF mass spectrometer, and *m/z* values are reported in Daltons to four decimal places. FTIR was acquired using a Bruker Platinum-ATR. ^{1}H, ^{13}C and ^{19}F NMR spectra were recorded in deuterated solvents on a Bruker Avance 500, Bruker Avance 400 or Bruker Avance DPX 300. Chemical shifts are quoted in parts per million downfield of tetramethylsilane and referenced to residual solvent peaks ($CDCl_3$: ^{1}H = 7.26 ppm, ^{13}C = 77.16 ppm) and coupling constants (*J*) are reported to the nearest 0.1 Hz. The following abbreviations are used: s, singlet; d, doublet; t, triplet, q, quartet; m, multiplet. Assignment of spectra was based on expected chemical shifts and coupling constants, aided by COSY, HMQC and HMBC measurements where appropriate.

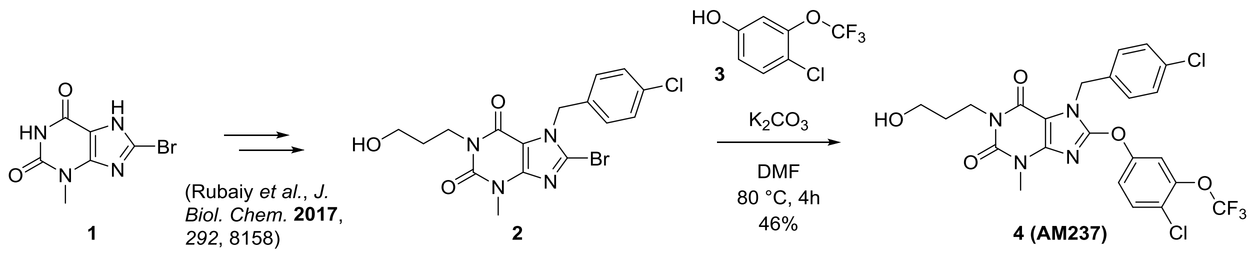

Figure S1. Synthetic route to AM237

8-[4-chloro-3-(trifluoromethoxy)phenoxy]-7-[(4-chlorophenyl)methyl]-1- (3-hydroxypropyl)-3-methyl-2,3,6,7-tetrahydro-1*H*-purine-2,6-dione (4, AM237)

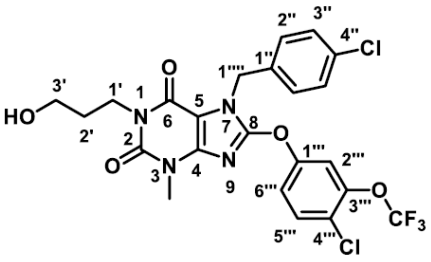

4-Chloro-3-(trifluoromethyl)phenol **3** (127 mg, 0.64 mmol) was added to a suspension of 8-bromo-7-[(4-chlorophenyl)methyl]-1-(3-hydroxypropyl)-3-methyl-2,3,6,7- tetrahydro-1*H*-purine-2,6-dione **2** (Hussein N Rubaiy et al. 2017) (250 mg, 0.59 mmol) and K_2CO_3 (162 mg, 1.17 mmol) in DMF (3.5 mL) and the reaction mixture was stirred at 80 °C for 4 hours. After cooling to room temperature, the reaction mixture was diluted with water (30 mL) and extracted with EtOAc (30 mL, then 3 × 20 mL). The combined organic layers were washed with aqueous LiCl solution (1M aq. solution 3 × 20 mL), brine (20 mL), dried (Na_2SO_4) and concentrated *in vacuo*. The crude material was purified by column chromatography (SiO_2; eluting with 2:8 EtOAc:hexanes) to yield the *title compound* as an off-white solid (152 mg, 0.27 mmol, 46%). *R*f 0.35 (2:8 EtOAc:hexanes); δH (400 MHz, $CDCl_3$) 7.51 (1H, d, *J* 8.9, 5’’’-C*H*), 7.40 (1H, d, *J* 2.8, 2’’’-C*H*), 7.38 (2H, d, *J* 8.4, 3’’-C*H*), 7.30 (2H, d, *J* 8.4, 2’’-C*H*), 7.20 (1H, dd, *J* 8.9, 2.8, 6’’’-C*H*), 5.41 (2H, s, 1’’’’-CH_2) 4.17 (2H, t, *J* 5.4, 1’-CH_2), 3.52 (2H, t, *J* 5.9, 3’-CH_2), 3.42 (3H, s, CH_3) 1.88 (2H, tt, *J* 5.9, 5.4 2’-CH_2); δC (100 MHz, $CDCl_3$) 155.3 (*C*-6), 152.6 (*C*-8), 151.6 (*C*-2), 146.2 (*C*-4), 145.4 (C-1’’’),134.7 (*C*-4’’), 134.1 (*C*-1’’), 131.4 (*C*H-5’’’), 129.9 (*C*-4’’’), 129.8 (*C*H-2’’), 129.3 (*C*H-3’’), 124.7 (*C*-5), 120.43 (q, *J* 260.3, CF_3), 119.2 (*C*H-6’’’),114.9 (*C*H-2’’’), 103.1 (C-3’’’), 58.7 (CH_2-1’), 46.8 (CH_2-1’’’’), 37.9 (CH_2-3’), 31.0 (CH_2-2’), 30.0 (CH_3) δF (235 MHz, $CDCl_3$) -57.9; νmax/cm-1 (neat); 3495, 1891, 1693, 1652; ESI-HRMS: calcd. for $C_{23}H_{20}{}^{35}Cl_2F_3N_4O_5$ $[M+H]^+$ 559.0757, found 559.0766. HPLC: RT = 1.99 min.

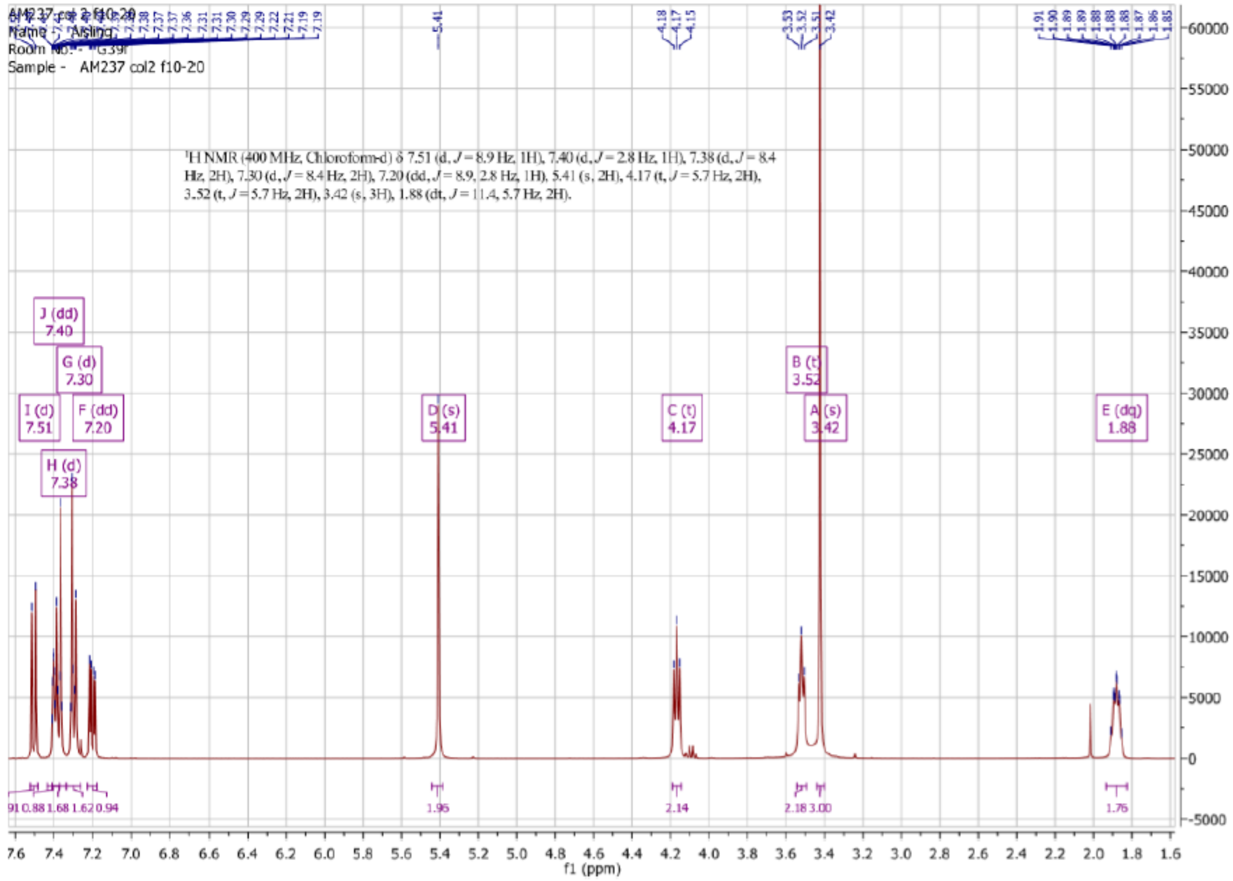

Figure S2. ^{1}H NMR spectrum of AM237

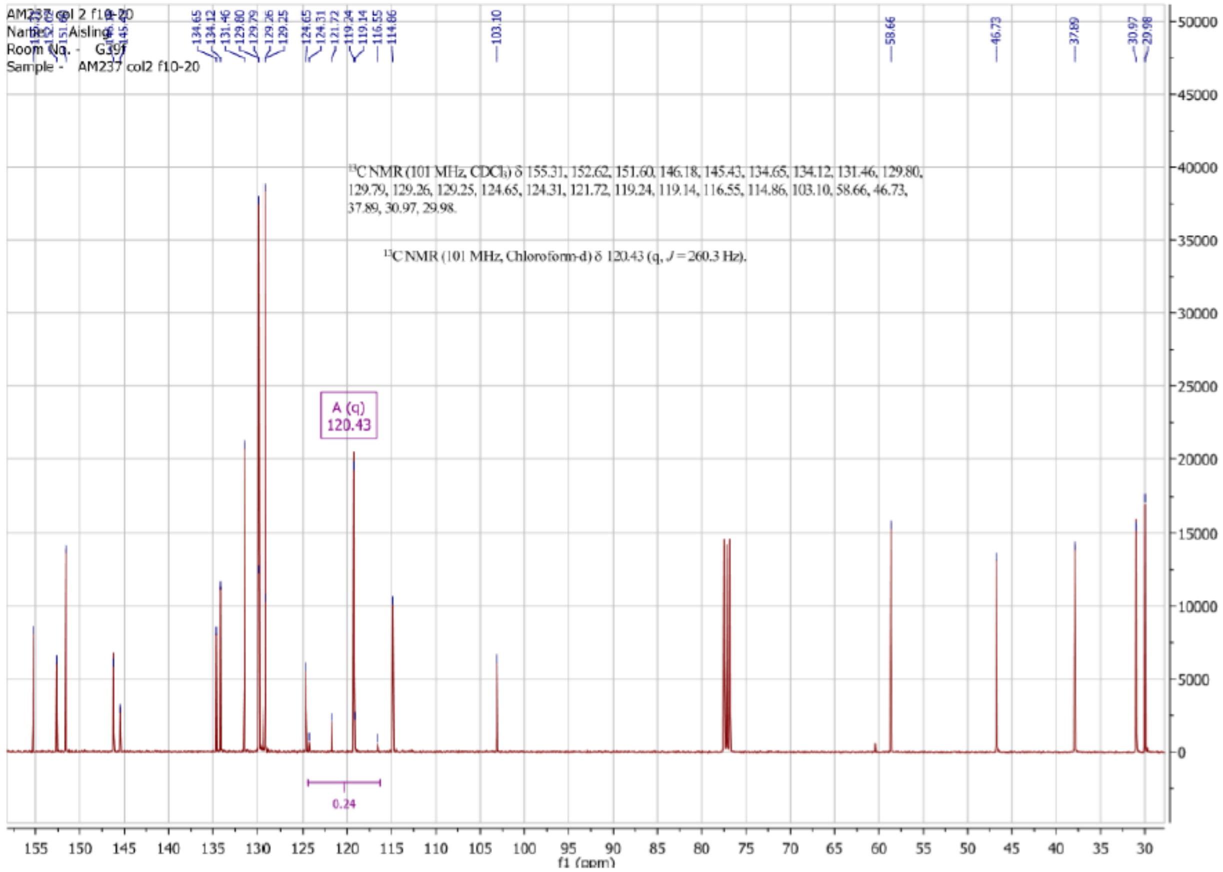

Figure S3. ^{13}C NMR spectrum of AM237

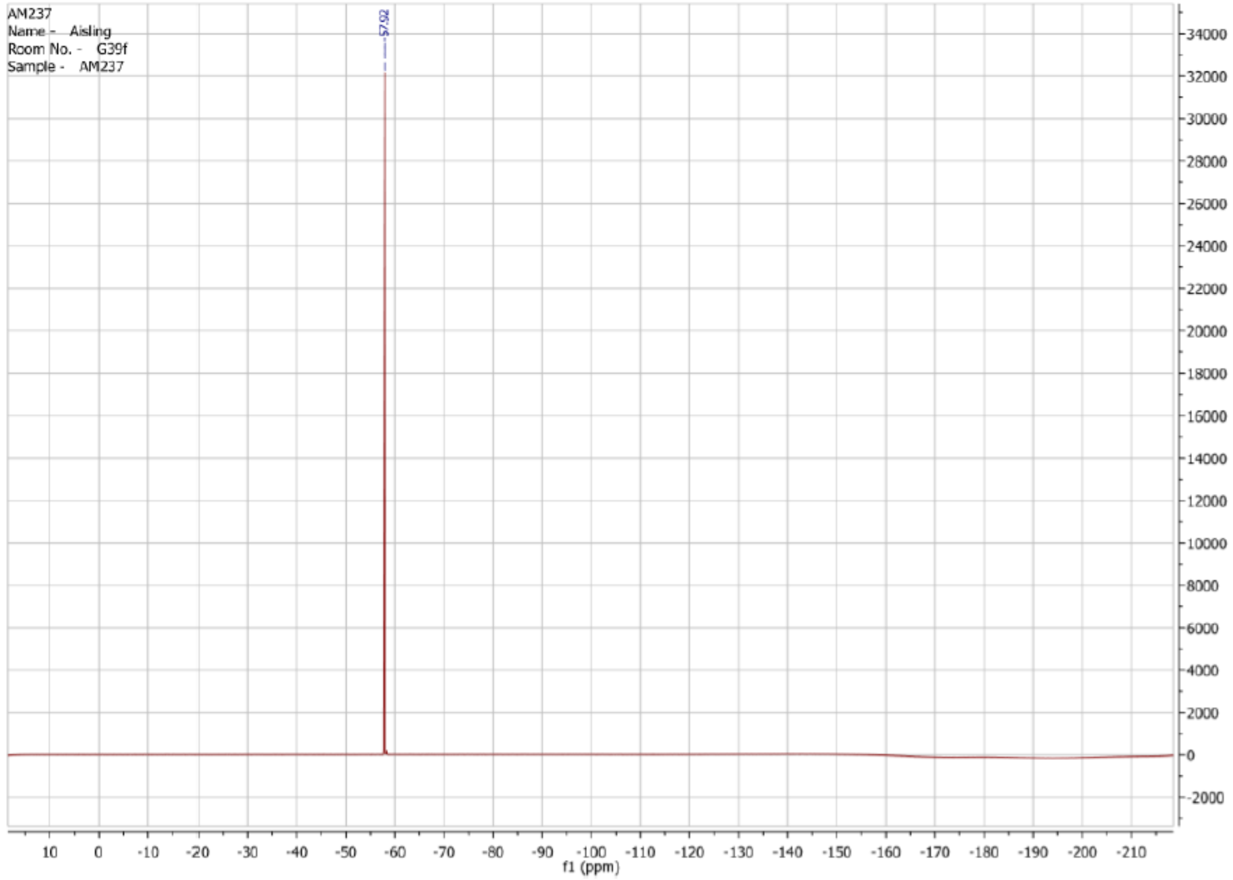

Figure S4. ^{19}F NMR spectrum of AM237

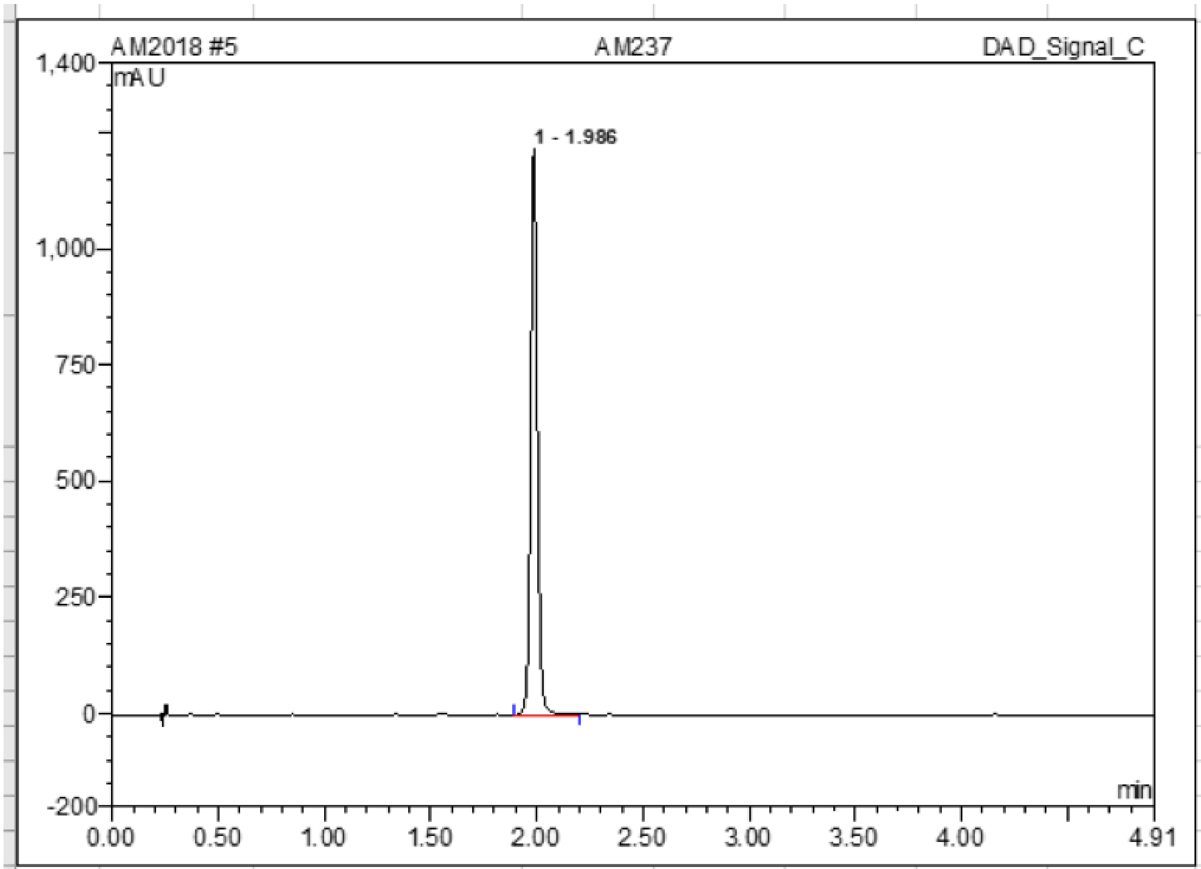

No.	Ret.Time min	Peak Name	Height mAU	Area mAU*min	Rel.Area %	Amount	Type
1	1.99	n.a.	1218.831	45.200	100.00	n.a.	BMB
Total:			1218.831	45.200	100.00	0.000	

Figure S5. HPLC chromatogram of AM237

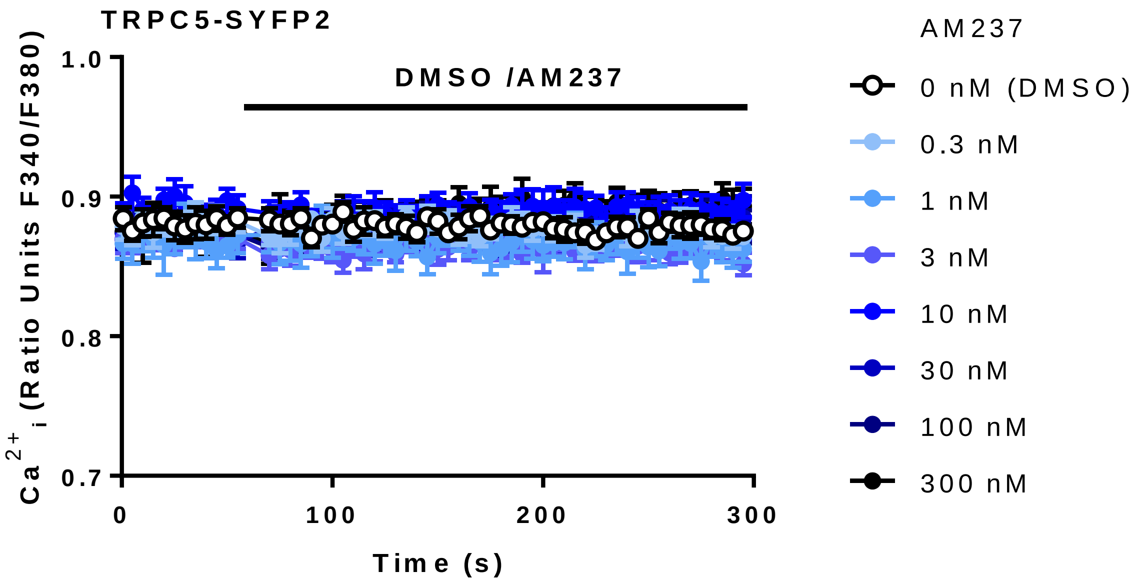

Figure S6. AM237-mediated increase of $[Ca^{2+}]_i$ is dependent on the presence of extracellular calcium. A) $[Ca^{2+}]_i$ measurements from a single 96-well plate (N = 6) showing that 0.3-300 nM AM237 has no effect on $[Ca^{2+}]_i$ in TRPC5-SYFP2 expressing (Tet+) HEK T-REx cells in the absence of extracellular calcium. Both recording buffer and compounds were made up in Ca^{2+}-free SBS, where $CaCl_2$ was replaced by 0.4 mM EGTA.

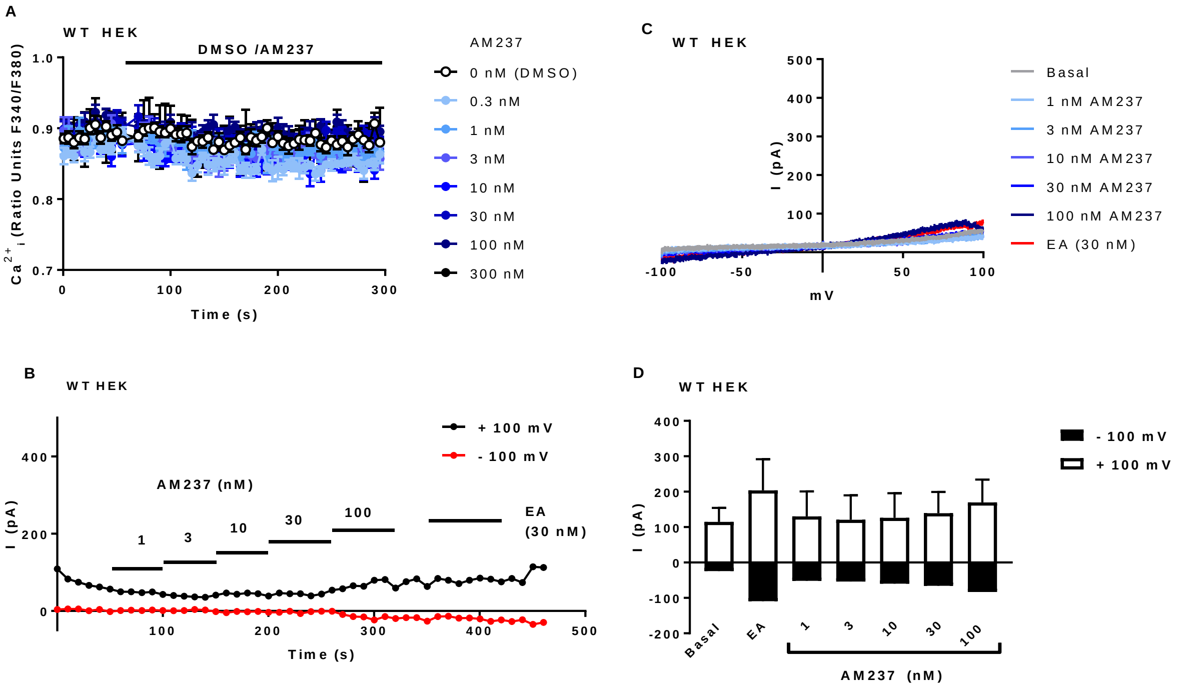

Figure S7. AM237-mediated increase of $[Ca^{2+}]_i$ is dependent on the expression of TRPC5 . A) $[Ca^{2+}]_i$ measurements from a single 96-well plate (N = 6) showing that 0.3-300 nM AM237 has no effect on $[Ca^{2+}]_i$ in wild-type HEK 293 cells. B) Example whole-cell patch clamp data from a WT HEK 293 cell showing current sampled at -100 mV and +100 mV during ramp changes in voltage. AM237 and EA had no significant effect on currents recorded. C) Representative current-voltage relationship (I-Vs) from experiments of the type illustrated in (B). D) Mean response ± SEM (n = 5/6 independent recordings) as illustrated in (B) for +100 mV and -100 mV.

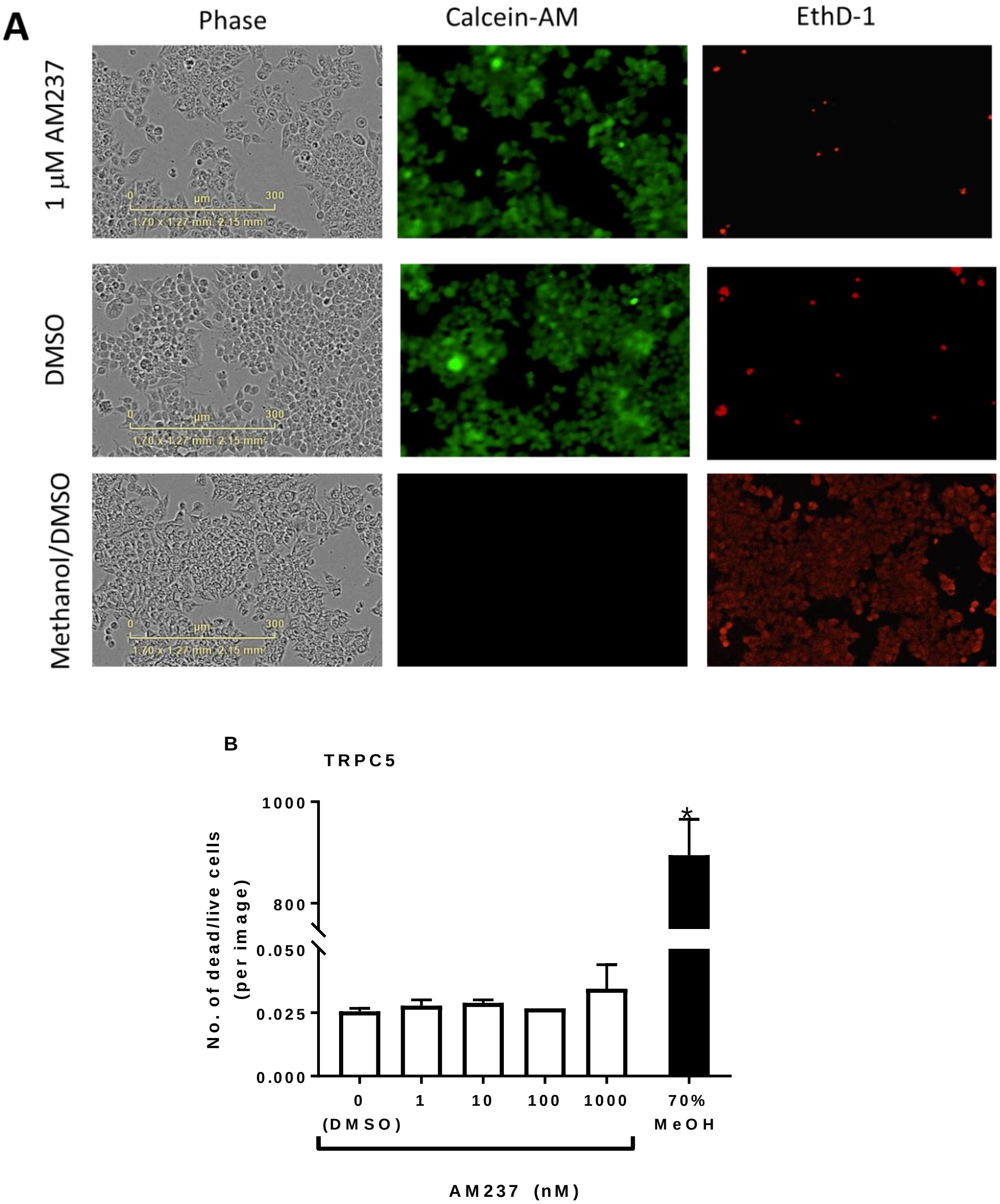

Figure S8. AM237 has no effect on cell viability of HEK T-REx (Tet+) expressing TRPC5. A) Representative images from LIVE/DEAD® cell viability assay for HEK T-REx (Tet +) cells expressing TRPC5 treated with AM237 (1 µM), DMSO (vehicle control) or methanol (positive control). Column 1, phase image of HEK T-REx cells expressing TRPC5. Column 2, Calcein AM staining for live cells. Column 3, Ethidium homodimer-1 (EthD-1) staining for dead cells. Row 1, cells treated with 1 mM AM237. Row 2, cells treated with DMSO. Row 3, cells fixed with methanol prior to treatment with DMSO. B) Mean ± SEM from experiment in (A). Data from three independent experiments.

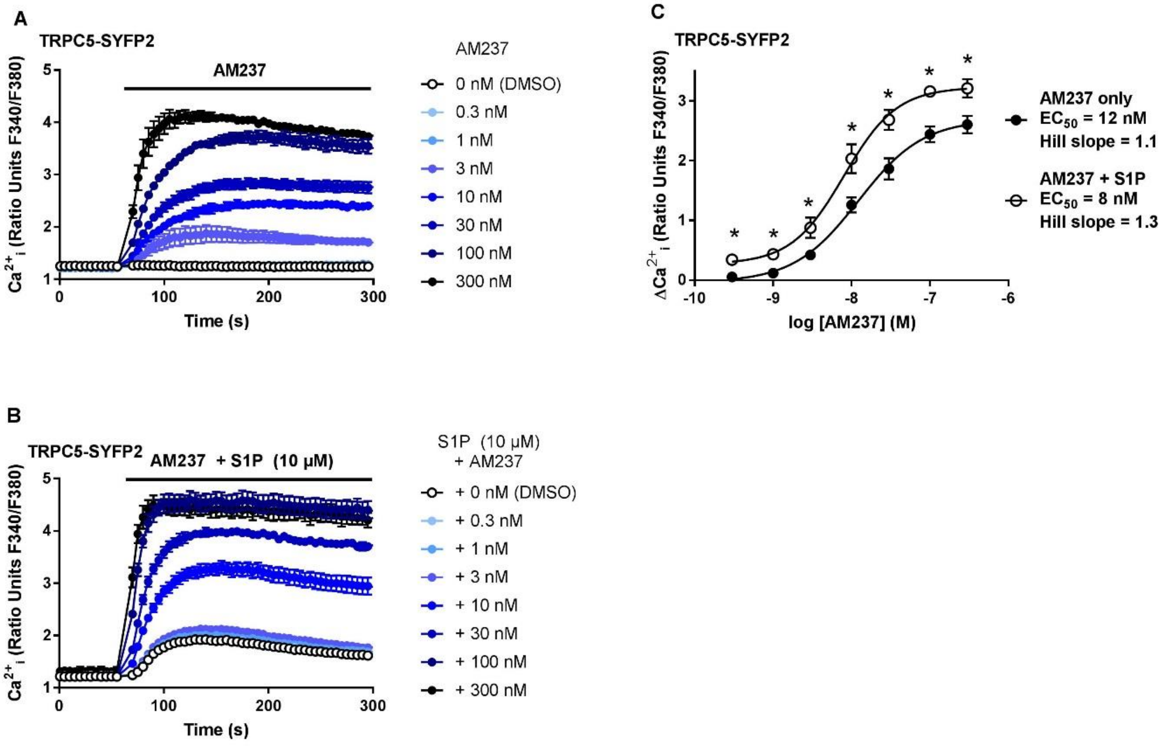

Figure S9. AM237 potentiates the S1P response of TRPC5:C5 channels. A) Representative $[Ca^{2+}]_i$ measurements from a single 96-well plate (N = 6) showing an increase in $[Ca^{2+}]_i$ in response to 0.3-300 nM AM237 in hTRPC5-SYFP2 expressing (Tet+) HEK T-REx cells. B) Representative $[Ca^{2+}]_i$ measurements from a single 96-well plate (N = 6) showing an increase in $[Ca^{2+}]_i$ upon co-application of 10 µM S1P and 0.3-300 nM AM237 in hTRPC5-SYFP2 expressing (Tet+) HEK T-REx cells. C) Concentration-response data for experiments in (F) and (G), showing mean responses ± SEM (n/N = 5/30). Responses were calculated at 250-295 s compared to $[Ca^{2+}]_i$ at baseline (0-55 s).

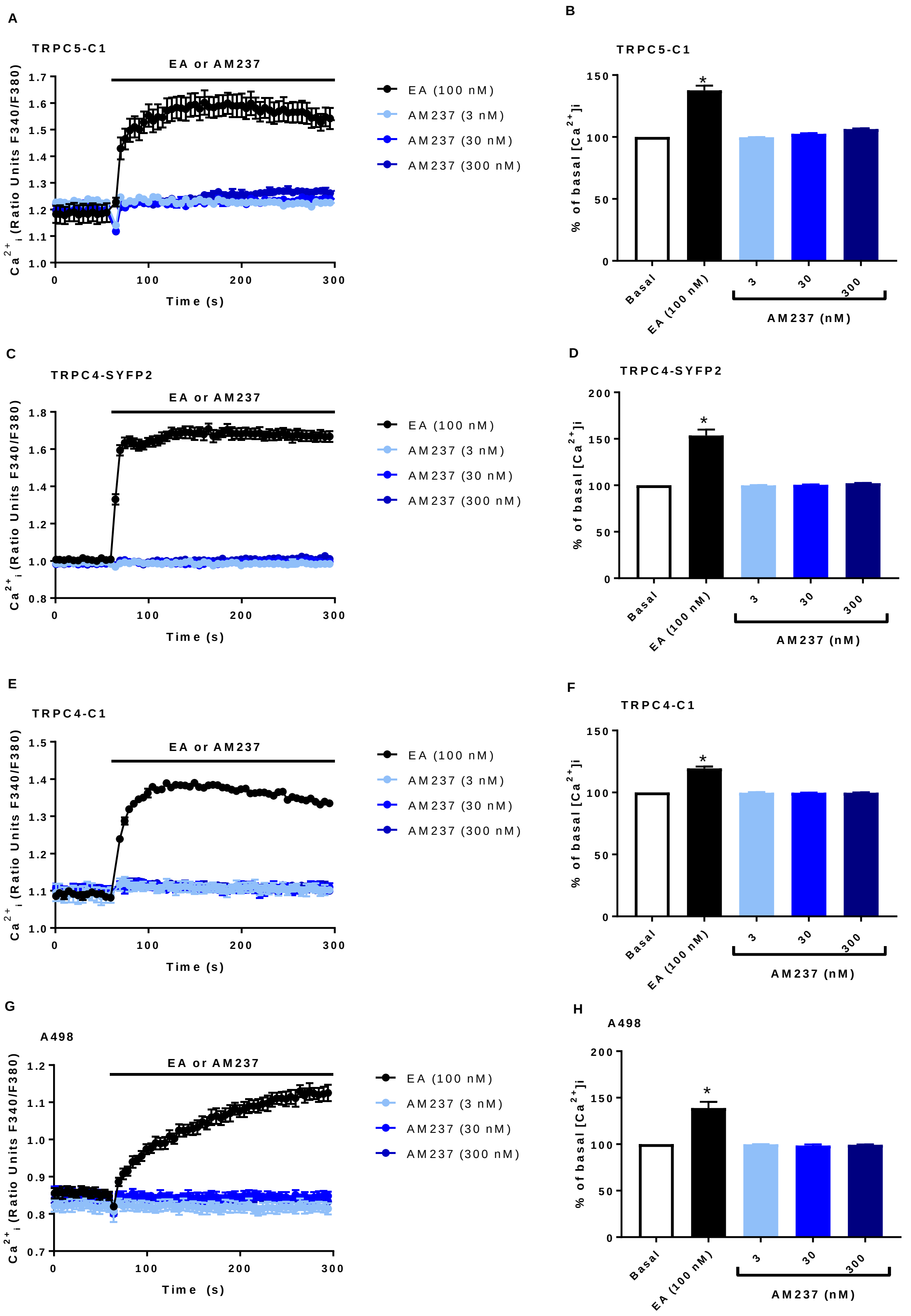
A
TRPC5-C1
EA or AM237
Ca2+i (Ratio Units F340/F380)
Time (s)
EA (100 nM)
AM237 (3 nM)
AM237 (30 nM)
AM237 (300 nM)
B
TRPC5-C1
% of basal [Ca2+]i
Basal
EA (100 nM)
AM237 (nM)
C
TRPC4-SYFP2
D
TRPC4-SYFP2
E
TRPC4-C1
F
TRPC4-C1
G
A498
H
A498

Figure S10. AM237 does not activate TRPC5:C1, TRPC4 and TRPC4:C1 channels A, C, E) Representative $[Ca^{2+}]_i$ measurements from single 96-well plates (N = 6) showing an increase in $[Ca^{2+}]_i$ in response to 100 nM EA, and lack of activation with 3-300 nM AM237 in (Tet+) HEK T-REx cells expressing hTRPC5-C1 (A), hTRPC4-SYFP2 (C) and hTRPC4-C1 (E). B, D, F) Mean ± SEM for experiments in (A), (C), and (E) respectively. Responses were calculated at 250-295 s compared to basal $[Ca^{2+}]_i$ 0-55 s (n/N = 3/18) G) Representative $[Ca^{2+}]_i$ measurements from a single 96-well plate (N = 6) showing an increase in $[Ca^{2+}]_i$ in response to 100 nM EA, and lack of activation with 3-300 nM AM237 in A498 cells. H) Mean ± SEM for experiments in (G). Responses were calculated at 250-295 s compared to basal $[Ca^{2+}]_i$ 0-55 s (n/N = 3/18)

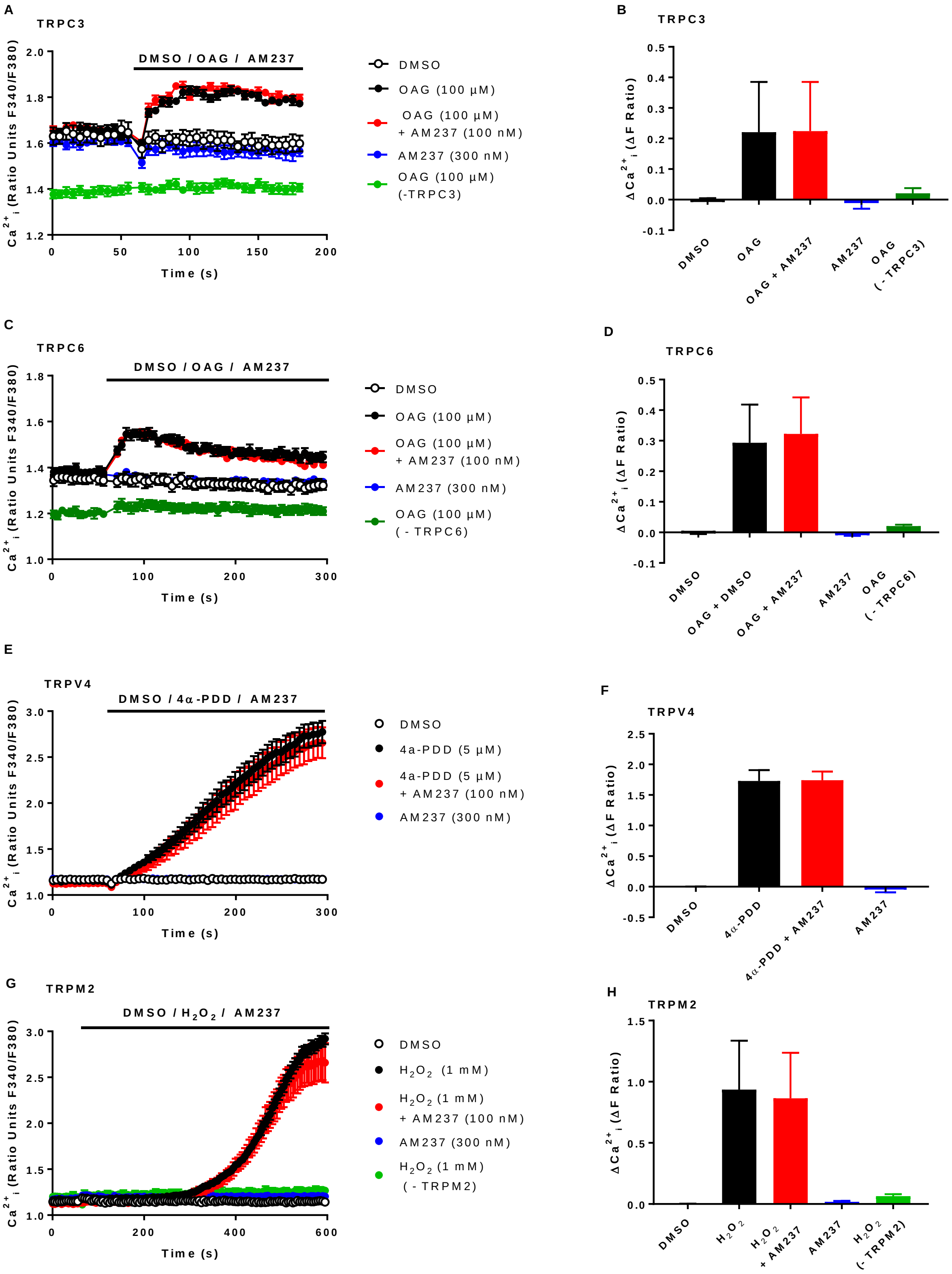

Figure S11. AM237 has no effect on TRPC3, TRPC6, TRPV4 or TRPM2 channels. A, C)

Representative $[Ca^{2+}]_i$ measurements from single 96-well plates (N = 6) showing no effect of AM237 on activation of hTRPC3 (A) or hTRPC6 (C) by 100 µM OAG in WT HEK 293 cells transiently transfected with hTRPC3 (A) or hTRPC6 (C). The baselines before OAG application indicate that AM237 does not activate TRPC3 (A) or TRPC6 (C). Cells not expressing TRPC3 (A) or TRPC6 (C) do not respond to 100 µM OAG. B,D) Mean responses ± SEM (n/N = 3/18) for experiments shown in (A,C), for hTRPC3 (B) and hTRPC6 (D). Responses were calculated at 75-95 s (TRPC3) and 80-100 s (TRPC6) compared to basal $[Ca^{2+}]_i$ (0-55 s). E) Representative $[Ca^{2+}]_i$ measurements from a single 96-well plate (N = 6) showing no effect of AM237 on activation of TRPV4 by 5 µM 4α-PDD in CHO cells stably expressing hTRPV4. The baselines before 4α-PDD application indicate that AM237 does not activate TRPV4. F) Mean responses ± SEM (n/N = 3/18) for experiments shown in (E). Responses were calculated at 250-295 s compared to basal $[Ca^{2+}]_i$ (0-55 s). G) Representative $[Ca^{2+}]_i$ measurements from a single 96-well plate (N = 6) showing no effect of AM237 on activation of TRPM2 by 1 mM H_2O_2 in (Tet+) HEK T-REx cells expressing TRPM2. The baselines before H_2O_2 application indicate that AM237 does not activate TRPM2. Cells not expressing TRPM2 do not respond to H_2O_2. H) Mean responses ± SEM (n/N = 3/18) for experiments shown in (F). Responses were calculated at 550-595 s compared to basal $[Ca^{2+}]_i$ (0-55 s).

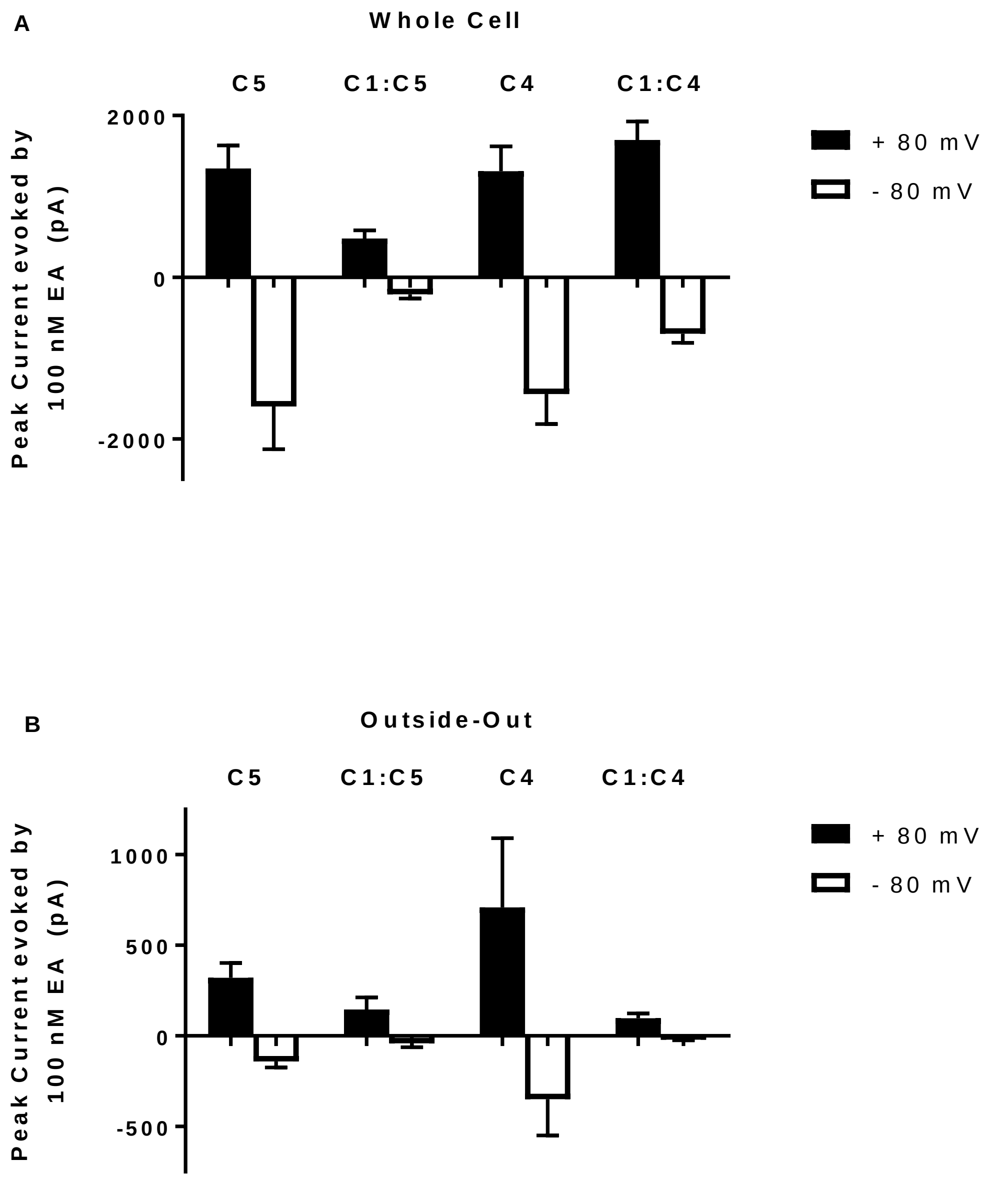

Figure S12. Differences in current amplitudes of TRPC5:C5, TRPC5:C1, TRPC4:C4 and TRPC4:C1 channels recorded by whole-cell patch and outside-out patch clamp electrophysiology. A) Mean ± SEM peak currents evoked by 100 nM EA in HEK 293 cells expressing either TRPC5, TPRC1 and TRPC5, TRPC4, or TRPC1 and TRPC4, measured by whole-cell patch clamp electrophysiology, with current sampled at -80 mV (white bars) and +80 mV (black bars) (n = 4-12). B) Mean ± SEM peak currents evoked by 100 nM EA in HEK 293 cells expressing either TRPC5, TPRC1 and TRPC5, TRPC4, or TRPC1 and TRPC4, measured in excised outside-out membrane patches, with current sampled at -80 mV (white bars) and +80 mV (black bars) (n = 4-8). Note the difference in scale of the y-axes between (A) and (B).
